# Metabolism and Epigenetic Interplay in Cancer: Regulation and Putative Therapeutic Targets

**DOI:** 10.3389/fgene.2018.00427

**Published:** 2018-10-09

**Authors:** Vera Miranda-Gonçalves, Ana Lameirinhas, Rui Henrique, Carmen Jerónimo

**Affiliations:** ^1^Cancer Biology and Epigenetics Group, Research Center (CI-IPOP), Portuguese Oncology Institute of Porto, Porto, Portugal; ^2^Master in Oncology, Institute of Biomedical Sciences Abel Salazar (ICBAS), University of Porto, Porto, Portugal; ^3^Department of Pathology, Portuguese Oncology Institute of Porto, Porto, Portugal; ^4^Department of Pathology and Molecular Immunology, Institute of Biomedical Sciences Abel Salazar (ICBAS), University of Porto, Porto, Portugal

**Keywords:** cancer metabolism, metabolites, DNA methylation, histone modifications, epigenetic plasticity

## Abstract

Alterations in the epigenome and metabolism affect molecular rewiring of cancer cells facilitating cancer development and progression. Modulation of histone and DNA modification enzymes occurs owing to metabolic reprogramming driven by oncogenes and expression of metabolism-associated genes is, in turn, epigenetically regulated, promoting the well-known metabolic reprogramming of cancer cells and, consequently, altering the metabolome. Thus, several malignant traits are supported by the interplay between metabolomics and epigenetics, promoting neoplastic transformation. In this review we emphasize the importance of tumour metabolites in the activity of most chromatin-modifying enzymes and implication in neoplastic transformation. Furthermore, candidate targets deriving from metabolism of cancer cells and altered epigenetic factors is emphasized, focusing on compounds that counteract the epigenomic-metabolic interplay in cancer.

## Epigenetic Mechanisms in Cancer: a Brief Overview

Chromatin results from the macromolecular complex of DNA and histone proteins, constituting the scaffold for packaging the entire genome. Chromatin is mostly composed of highly condensed regions, replicating late and containing inactive genes (heterochromatin) and decondensed regions, containing most of the active genes (euchromatin) (Dawson and Kouzarides, 2012). These distinct conformations are responses to appropriate intrinsic and extrinsic signals, responsible for altering gene activity and cellular phenotype. During normal development, chromatin stability is imposed to a restrictive state that blocks differentiation programs. Yet, genetic [eg., Polycomb EZH2 (McCabe et al., 2012) and isocitrate dehydrogenase (IDH) (Yan et al., 2009) mutations], environmental [e.g., hypoxia (Thienpont et al., 2016) and inflammation (O’Keefe, 2016)], or metabolic [butyrate (O’Keefe, 2016), folate (Waterland and Jirtle, 2003), and vitamin C (Hore et al., 2016)] insults can induce overly restrictive or overly permissive epigenetic landscape that contributes to neoplastic transformation (Flavahan et al., 2017). Restrictive chromatin states may prevent appropriate induction of tumour suppressor programs or block differentiation. The homeostatic chromatin network is predicted by an interplay between repressors, activators and remodelers (Piunti and Shilatifard, 2016). Thus, epigenetics corresponds to heritable traits not involving alterations in DNA sequence, but rather chemical changes within the chromatin.

The information conveyed by epigenetic modifications plays a critical role in the regulation of all DNA-based processes, including transcription, DNA repair, and replication. Consequently, abnormal expression patterns or genomic alterations in chromatin regulators may have profound effects in cell homeostasis and can lead to cancer initiation and/or progression (Dawson and Kouzarides, 2012). Indeed, epigenetic deregulation may precede transforming genetic events, including mutations in tumor suppressors and/or proto-oncogenes, and genomic instability (Rodriguez-Paredes and Esteller, 2011; Dawson and Kouzarides, 2012). Furthermore, some studies showed that cancer portrays a catalog of recurrent somatic mutations in several epigenetic regulators (Dawson and Kouzarides, 2012).

**FIGURE 1:**
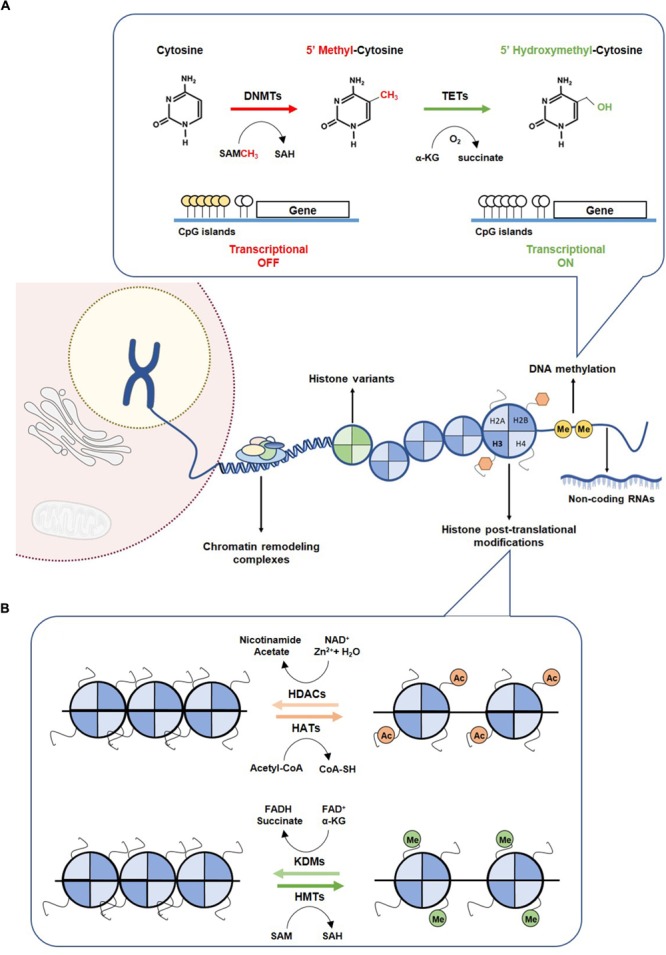
Epigenetic mechanism. **(A)** DNA methylation mechanism. Methylation of cytosine to 5-methylcytosine is catalyzed by DNMTs, through the methyl donor SAM, which is converted to SAH. Hypermethylation of CpG islands of promoter regions leading to transcriptional gene repression. Hydroxylation of 5mC to 5hmC by TETs promotes a transcriptional gene activation. **(B)** Histone modifications. Covalent modification on histones control the accessibility of DNA to transcription factors. The writers HATs and HMTs sign acetylated and methylated marks using as co-factors the acetyl-CoA and SAM, respectively. Acetylated and methylated markers can be removed by erasers, such as HDACs and KDMs, using as co-factor Zn2+ and NAD^+^, respectively. DNMTs, DNA methyltransferases; HATs, histone acetyltransferases; HDACs, histone deacetylases; HMTs, histone methyltransferases; KDMs, histone demethylases; SAH, S-adenosylhomocysteine; SAM, S-adenosylmethionine; TETs, Ten-eleven translocation family.

The most studied epigenetic alterations associated with neoplastic phenotype are variation in DNA methylation, alterations in histone proteins structure through post-translational modifications and histone variants (Figure 1) (Biswas and Rao, 2017). Additionally, microRNAs, which are small RNA molecules (22 nucleotides long), post-transcriptionally control gene expression (Chuang and Jones, 2007). miRNAs’ expression is dynamic, acting in several cellular pathways, and one single miRNA can target multiple genes whereas several miRNA can target the same gene (Gambari et al., 2011; Zhang et al., 2012). Indeed, miRNAs, can function as oncogenes or tumor suppressors (Rupaimoole and Slack, 2017), impacting on metabolic pathways, including glutaminolysis, glycolysis and Krebbs cycle (Chuang and Jones, 2007; Chen et al., 2012).

### DNA Methylation

Cytosine methylation occurring in regions with high frequency of CpG sites (CpG islands), mostly residing at promoter regions, is strongly implicated in transcriptional silencing (Easwaran et al., 2014). The addition of a methyl group to cytosine is mediated by DNA methyltransferases (DNMT1, DNMT3a and DNMT3b) using a methyl donor *S*-adenosyl-L-methionine (SAM). DNMT1 preferentially methylates the unmethylated stand of hemi-methylated DNA during replication, whereas DNMT3a and DNMT3b catalyze *de novo* methylation in both strands (Li et al., 1992). DNA demethylation involves oxidation of 5mC, catalyzed by enzymes of the ten-eleven translocation protein (TET) family to generate 5hmC (Figure 1A) (Tahiliani et al., 2009). In normal cells, CpG islands are mostly unmethylated, whereas CG-poor regions within genes bodies are highly methylated (Easwaran et al., 2014). Many cancers, however, display distinct shifts in DNA methylation patterns toward hypermethylation at CpG islands and hypomethylation within gene bodies (Easwaran et al., 2014). The most widely recognized epigenetic disruption in human cancers is CpG island promoter hypermethylation-associated silencing of tumor suppressor genes such as CDKN2A, MLH1, BRCA1 and VHL, which has been identified as driver for lung, colorectal, breast and renal cancer progression (Jones and Baylin, 2007; Esteller, 2008). Briefly, CDKNA methylation has been associated with increased SETDB1 expression and consequently uncontrolled tumor cell proliferation (Zhao et al., 2016) and the well-recognized MLH1 promoter hypermethylation in colorectal cancers seems to result from increased H3K9me3 levels through LSD1 activity, which consequently favors the glycolytic metabolism in hypoxic conditions (Lu et al., 2014). Recently, BRCA1 methylation and consequent reduced expression have been associated with increased glycolytic metabolism and tumor progression (Privat et al., 2014). Moreover, in clear cell renal cell carcinoma, absence of VHL expression, due to VHL mutation and/or promoter hypermethylation, leading to HIF-1α constitutive activation, was associated with increased glycolytic metabolism (Semenza, 2007). Both mutations and epimutations have been found in genes encoding for enzymes involved in establishment and/or removal of specific DNA methylation patterns (Plass et al., 2013).

### Histone Modifying Enzymes

Histone tails are marked for multiple modifications which are recognized by reader proteins that sequentially translate the information into distinct transcriptional profiles through alterations of chromatin states. Both histone modifications and their readers, namely polycomb complex (PC) binding to H3K27me3 and heterochromatin protein 1 (HP1) binding to H3K9me3/2, determine whether chromosomal regions are accessible for binding of transcription factors or other regulatory molecules. These specific modifications of particular amino-acids motifs of core histones are established by histone acetyltransferases (HATs) or histone methyltransferases (HMTs) that can be removed by histone deacetylases (HDACs) and histone lysine demethylases (KDMs), respectively (Figure 1B) (Bannister and Kouzarides, 2011). Acetylation of histone residues facilitates gene transcription by loosening chromatin compaction or enhancing transcriptional activators recruitment. Genome-wide analyses showed a strong enrichment of histone acetylation at promoters and enhancers of active genes, namely acetylation of H3K27 (Wang et al., 2008). Indeed, in cancer cells, pathological activation of tumorigenic enhancers was associated with H3K27ac aberrant accumulation (Djebali et al., 2012). Chromosomal translocations involving HATs’ encoding genes, namely EP300 and CREBBP have been identified in hematological cancers (Yang, 2004). Additionally, a missense mutation in EP300 has been found in colorectal, gastric, breast and pancreatic tumors, whereas monoallelic loss of KAT5 increases malignant transformation (Gayther et al., 2000).

Unlike histone acetylation, histone methylation is site-specific and chromatin context-dependent. H3K4 (di and tri) methylation is strongly associated with active transcription, whereas H3K27 methylation is involved in transcriptional repression (Bernstein et al., 2006). H3K4 and H3K27 methylation have been found in bivalent domains, namely H3K4me3 and H3K27me3, in several cancer cells and suggested to promote plasticity and tumors’ adaptation to different environments (Harikumar and Meshorer, 2015). Mutations in genes encoding these enzymes have been implicated in cancer, including MLL, an H3K4me3 HMT that associates with poor prognosis in AML (Krivtsov and Armstrong, 2007). Moreover, SMYD3, another H3K4 HMT, frequently upregulated in prostate, colorectal and hepatocellular carcinoma increases cell growth and promotes transformation (Hamamoto et al., 2004; Vieira et al., 2015). Additionally, EZH2, an H3K27 HMT is overexpressed in several solid tumors, such as prostate, breast, colon, skin and lung cancers (Bracken and Helin, 2009). Furthermore, overexpression and/or loss of function mutations in KDMs is believed to contribute for tumorigenesis in several cancer types. Genetic mutations of KDM5A and KDM5C, affecting H3K4 methylation, and KDM6A affecting H3K27 methylation, have been demonstrated in some cancer cells (Imielinski et al., 2012; Jones et al., 2012; Vieira-Coimbra et al., 2015).

Gain-of-function EZH2 mutations are frequent in several lymphoma subtypes and melanoma leading to expansive H3K27me3, which appears to induce a repressive state that prevents induction of differentiation genes (McCabe et al., 2012). The KDMs have also been implicated in cancer, being upregulated under stress conditions and in response to signals from tumor microenvironment (Black et al., 2012). Cancer-associated deregulation of these enzymes may confer plasticity and facilitate reprogramming for a permissive state (Black et al., 2015).

Gene expression regulation by epigenetics mechanisms is very adaptative to environmental factors (Feil and Fraga, 2012). As cancer cells divide, acquired epigenetics states may be maintained through cell division by DNA methylation, repressive chromatin, or gene regulatory circuits, giving rise to adaptive epi-clones that fuel malignant progression (Flavahan et al., 2017). By contrast, permissive or plastic states may allow oncogene activation or non-physiologic cell fate transitions. This plasticity state may confer advantage for cancer cells and be selected as drivers. In mutated gliomas, particularly those with IDH mutation, chromatin structure destabilizes and, thereby, triggers epigenetic instability. Thus, hypermethylated phenotype associated to IDH mutant gliomas promote aberrant activation of platelet-derived growth factor receptor A (PDGFRA), which fosters uncontrolled proliferative signalling, a recognized hallmark of cancer (Flavahan et al., 2017).

## Tumor Metabolism

During cancer initiation and/or progression, molecular changes associated with metabolic reprogramming are needed to meet cancer cells energy demands, which often is coordinated with elevated biosynthetic processes and energy production (Vander Heiden et al., 2009), a recognized cancer hallmark (Hanahan and Weinberg, 2011).

In normal cells from quiescent tissues, glycolysis is reduced in the presence of oxygen and energy production arises from mitochondrial oxidative phosphorylation (OXPHOS), which oxidizes pyruvate to CO_2_ and H_2_O (Pasteur effect). Tumor cells, however, are highly glycolytic even in the presence of oxygen, constituting the major energy source (Warburg effect) (Warburg, 1956b; Gatenby and Gillies, 2004). Consequently, tumor cells convert most of incoming glucose into lactate rather than metabolizing pyruvate in mitochondria through OXPHOS (Warburg, 1956a; Gatenby and Gillies, 2004).

The glycolytic pathway is bioenergetically less efficient than OXPHOS, since glucose metabolism yields less ATP molecules compared to OXPHOS. However, metabolic reprogramming with increased glycolytic rates allows tumor cells to adapt to fluctuating oxygen availability conditions enabling fast ATP production due to high glucose uptake, as well as NADPH generation through pentose phosphate pathway (PPP) (Kroemer and Pouyssegur, 2008; Vander Heiden et al., 2009). Additionally, tumor cells may use glycolytic pathway intermediates for anabolic reactions, enabling biosynthesis of lipids, amino acids and nucleotides (Kroemer and Pouyssegur, 2008; Vander Heiden et al., 2009). The Warburg effect contributes to antioxidant glutathione production counteracting ROS and protecting cells from oxidative stress. Furthermore, it increases lactate production and consequent microenvironment acidification, which has been implicated in cancer cells’ aggressiveness through increased migration, invasion, metastization, immunosuppression and therapy resistance (Gillies et al., 2008; Liberti and Locasale, 2016).

Although Warburg hypothesis postulates that cancer cells adopt a glycolytic phenotype due to mitochondrial damage at OXPHOS level, mitochondria are functional in cancer cells and several metabolites are produced by TCA cycle for biosynthetic pathways (Bui and Thompson, 2006). These observations suggest that rather than being an adaptation to defect in mitochondrial respiration, the Warburg effect is a regulated metabolic state and may be beneficial during a time of increased biosynthetic demand (Pavlova and Thompson, 2016). Thus, reprogramming of carbon metabolism by proliferating cells demonstrates that Warburg effect is an alternative for generation of intermediate metabolites in biosynthesis. Moreover, tumor cells increase anabolism, including nucleotide biosynthesis, through intermediates of glycolytic pathway, like glucose-6-phosphate (G6P) and fructose-6-phosphate (F6P), for ribose 5-phosphate production.

Along with glucose, glutamine is a source of energy for biosynthetic processes, functioning as a nitrogen donor (Reitzer et al., 1979; Pavlova and Thompson, 2016). Glutamine metabolism has been reported to be upregulated in some tumors, being crucial for several biosynthetic processes, including cholesterol, fatty acids and protein synthesis (Martinez-Outschoorn et al., 2017). Shift to glutamine metabolism produces acetyl-coenzyme A (acetyl-CoA), a lipid biosynthesis precursor, which is an adaptive mechanism as glycolytic metabolism prevents entry of pyruvate into mitochondria by pyruvate dehydrogenase kinase (PDK) overexpression (Daye and Wellen, 2012).

**FIGURE 2:**
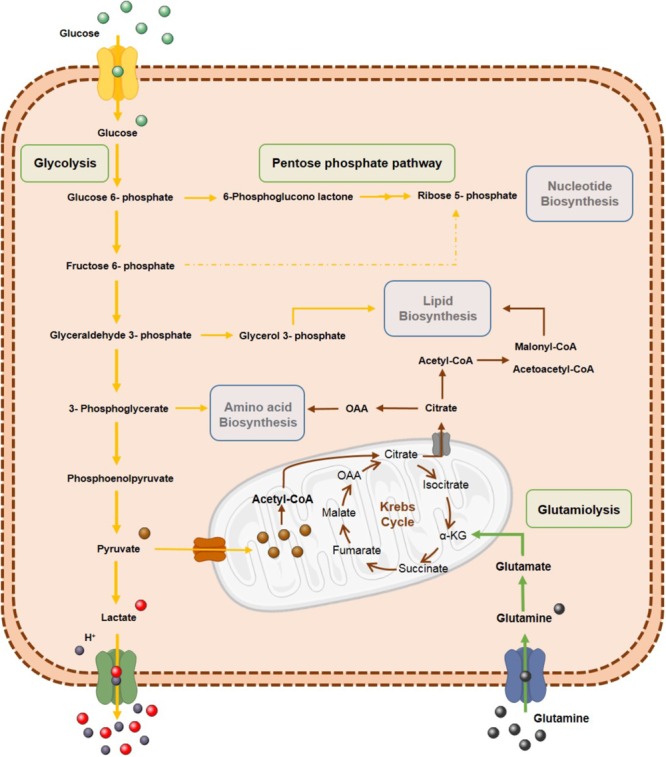
Tumor cells’ metabolism reprogramming. Metabolic reprogramming of tumor cells is characterized by highly glycolytic rates to increase tumor biomass by biosynthetic process. Glycolytic intermediates are used in pentose phosphate pathway (PPP) for anabolic process such as biosynthesis of nucleotides, amino acids and lipids. Glutamine is also an important source for tumor cells through glutaminolysis.

Tumor cell metabolic reprogramming also affects lipid biosynthesis. Indeed, *de novo* fatty acid synthesis occurring in tumor cells is important for cellular membrane biogenesis and energy storage (Menendez and Lupu, 2007). Furthermore, upregulation of several enzymes associated with lipogenesis, including acetyl-CoA carboxylase (ACC) and fatty acid synthase (FAS), has been reported in several neoplasms (Abramson, 2011). Additionally, lipogenesis may support tumor cell growth within nutrient-limited areas. Therefore, different metabolic pathways are reprogrammed to supply important metabolites for anabolic processes in response to different stimuli and stress conditions favoring tumor growth and progression (Figure 2).

Metabolic rewiring in cancer profoundly affects gene expression regulation. Although metabolite profiles profoundly impact epigenetic regulation, although genetic impact is minimal (Reid et al., 2017). Therefore, epigenetic and metabolic alterations in cancer cells are closely mechanistically linked. The accessibility to epigenetic enzymes’ co-factors might be altered due to reprogramming of cell metabolism, which gives rise to metabolic by-products that affect enzymatic activity, altering the epigenetic profile of cancer cells (Sharma and Rando, 2017). Moreover, metabolism is affected by altered expression of key enzymes due to epigenetic changes, impacting on control of several metabolic pathways (Wong et al., 2017). Thus, several malignant traits are supported by the interplay between metabolomics and epigenetics, promoting neoplastic transformation. An integrative comprehension of epigenetic and metabolic interplay in cancer is far from complete, but conceptual schemes are starting to emerge.

## Epigenetic Regulation of Metabolic Enzymes in Cancer

**FIGURE 3:**
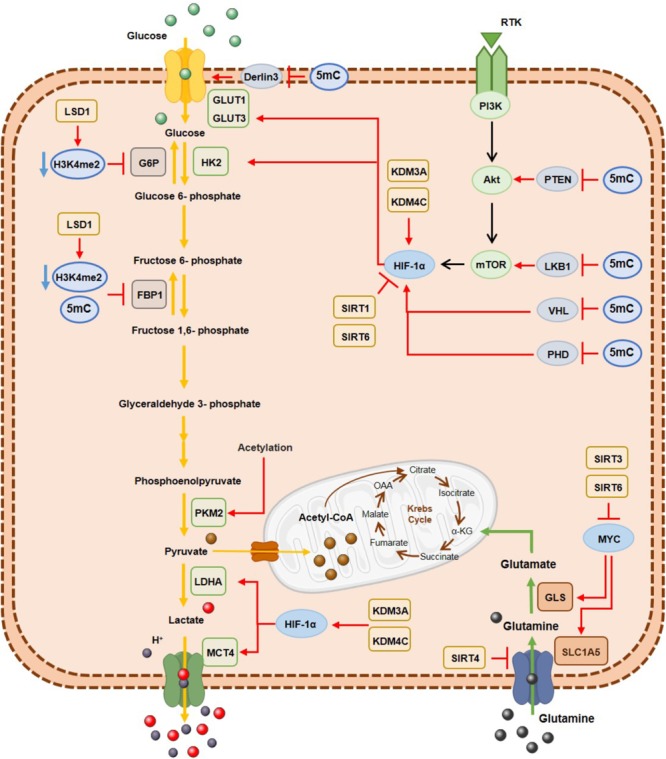
DNA hypermethylation, histone demethylases and histone deacetylases effect on the metabolic enzymes expression involved in glycolysis and glutaminolysis. Epigenetic mechanisms are associated with a metabolic cancer cell reprogramming by transcriptional repression of gluconeogenic enzymes and consequently activation of glycolytic pathway and glutaminolysis, where myc and HIF-1α transcription factors are important modulators. G6P, glucose 6-phospatase; GLS, glutaminase; GLUT1, glucose transporter 1; GLUT3, glucose transporter3; FBP1, fructose 1,6-biphosphatase; HK2, hexokinase2; LDHA; lactate dehydrogenase A; MCT4, monocarboxylate transporter 4; PKM2, pyruvate kinase M2; SLC1A5, glutamine transporter ASCT2.

Along with genetic mutations, epigenetic alterations are also a cause of metabolic enzymes deregulation in cancer cells (Figure 3) (Kaelin and McKnight, 2013). Specifically, hexokinase 2 (HK2) upregulation in hepatocellular carcinoma and glioblastoma results from promoter hypomethylation, favoring glycolytic flux (Goel et al., 2003; Wolf et al., 2011). Moreover, activity of acetylated pyruvate kinase M2 (PKM2) is reduced at the final glycolysis step, promoting glycolytic intermediates availability to biosynthetic process of nucleic acids, lipids and amino acids needed for biomass increment in tumor cell proliferation (Lv et al., 2011). Furthermore, fructose 1,6-biphosphate (FBP1), regulating gluconeogenesis, is transcriptionally silenced by promoter hypermethylation, inducing higher glycolytic rates in gastric, colon and liver cancers (Chen et al., 2011). The same was demonstrated in renal clear cell carcinoma cell lines (Liu et al., 2010) and basal-like breast cancer (Dong et al., 2013), in which DNMTs were recruited by Snail through G9a and SUV39H1, two HMTs that establish H3K9me3, leading to aberrant methylation of FBP1 promotor (Dong et al., 2013; Li and Li, 2015). Moreover, NFκB by interacting with LSD1 and HDACs directly suppresses FBP1 expression by decreasing H3K4me2 levels at its promoter (Pan et al., 2013) and HDACs/NFκB interaction may also recruit DNMTs, resulting in FBP1 promoter hypermethylation and stable silencing (Li and Li, 2015).

Conversely, glucose transporter 1 (GLUT1) overexpression is due to derlin-3 promotor hypermethylation, implicated in GLUT1 proteasome degradation (Lopez-Serra et al., 2014). Moreover, metabolic reprogramming of cancer cells derives from oncogene activation. The PI3K/AkT/mTOR pathway together with MYC and HIF1α transcription factors have been implicated in glycolytic metabolism (Jang et al., 2013). Concomitantly, PTEN (Salvesen et al., 2001; Kang et al., 2002; Soria et al., 2002; Garcia et al., 2004; Alvarez-Nunez et al., 2006), VHL (Herman et al., 1994; Schmitt et al., 2009; Vanharanta et al., 2013) and PDH (Place et al., 2011), which repress this pathway, are epigenetically silenced through promoter methylation, resulting in PI3K/AkT/mTOR pathway constitutive activation.

Concerning histone modifications involvement in metabolic rewiring, several KDMs were found overexpressed in different solid tumors. In bladder cancer KDM3A overexpression has been associated with metabolic shift to glycolysis. KDM3A is recruited to glycolytic genes’ promoters including GLUT1, HK2, phosphoglycerate kinase 1 (PGK1), lactate dehydrogenase A (LDHA) and monocarboxylate transporter 4 (MCT4), together with HIF-1α, leading to H3K9me2 demethylation and consequent transcriptional activation (Wan et al., 2017). Additionally, in hypoxic conditions, increased H3K27ac biding on HIF-1α induces glucose transporter 3 (GLUT3) overexpression through KDM3A binding (Mimura et al., 2012). Additionally, KDM4C overexpression in breast cancer is associated with increased glycolytic metabolism through HIF-1α interaction (Luo et al., 2012) and LSD1 is implicated in gluconeogenesis inhibition through H3K4me2 demethylation, leading to FBP1 and G6P transcription repression (Pan et al., 2013) and favoring HIF-1α-dependent glycolytic metabolism, in hepatocellular carcinoma (Sakamoto et al., 2015). Furthermore, in oesophageal cancer, LSD1 knockdown leads to decreased extracellular acidification, increased oxygen consumption and glucose uptake (Kosumi et al., 2016).

Among HDACs, Sirtuins’ family (HDAC class III) has been the most extensively studied concerning cell metabolism regulation. Indeed, SIRT6 and SIRT3 have been implicated in glucose homeostasis regulation (Chalkiadaki and Guarente, 2015). Glycolytic metabolism and glutaminolysis depending on HIF1α and MYC, respectively, are abrogated by SIRT6 (Zhong et al., 2010; Sebastian et al., 2012). Accordingly, SIRT6 deletion, observed in different tumours, like colon, pancreatic and hepatocellular carcinomas, leads to increased H3K9ac levels resulting in glycolytic gene expression upregulation promoting cellular transformation and, consequently tumour growth and progression (Sebastian et al., 2012; Chalkiadaki and Guarente, 2015). Additionally, mitochondrial SIRT3 was also shown to regulate the glucose homeostasis in HIF1α-dependent manner (Bell et al., 2011). In fact, SIRT3 loss is associated with cellular metabolism shift towards enhanced glycolysis in cancer cells (Vander Heiden et al., 2009). Furthermore, SIRT4 suppresses tumour growth by repressing glutamine metabolism (Jeong et al., 2013). Specifically, SIRT4 overexpression inhibits glutamine utilization and proliferation by a MYC-dependent manner in human Burkitt lymphoma cells (Jeong and Haigis, 2015). Nevertheless, SIRT4 downregulation has been reported in several tumours, like bladder, gastric and breast cancer (Chalkiadaki and Guarente, 2015). Although less consistently than SIRT4, SIRT1 was also associated with tumour suppressor function in cellular metabolic regulation (Chalkiadaki and Guarente, 2015), repressing glycolytic metabolism, indirectly through HIF1α deacetylation and directly by inhibiting the glycolytic enzyme phosphoglycerate mutase 1 (PGAM1) through deacetylation (Lim et al., 2010). Interestingly SIRT1 also has been implicated in lipid metabolism regulation under tumour nutrient deprivation (Jeong and Haigis, 2015). Regarding SIRT2, both oncogene or tumour suppressor functions have been suggested, depending on the tumour context (Chen et al., 2013; McGlynn et al., 2014). SIRT2 deacetylases FOXO1, modulating glucose and lipid metabolism in cellular stress and caloric restriction conditions (Jeong and Haigis, 2015). Indeed, SIRT2 promotes gluconeogenesis by deacetylating the enzyme phosphoenolpyruvate carboxykinase (PEPCK) (Jiang et al., 2011). SIRT2 is also involved in tumour metabolism regulation through MYC stabilization by deacetylating H4K16ac (Liu et al., 2013). Thus, SIRT2 and MYC are implicated in tumour metabolism regulation of MYC-induced malignancies working as a positive feedback loop.

Considering the multiple histone modifications contributing to gene regulation, much remains to be understood concerning the role of histone code in cancer metabolic reprogramming. Recently, anabolic glucose metabolism was associated with distant metastasis during pancreatic ductal adenocarcinoma progression (McDonald et al., 2017). Throughout sub-clonal evolution, decreased HEK9me2a and H3K9me3 was coupled with increased H3K9ac, H3K27ac and H4K16ac marks. These epigenomic reprogramming paralleled increased glycolytic metabolism, namely by increased glucose uptake and lactate production, along with nucleotides synthesis through PPP.

## Metabolites and Cancer Epigenetic Landscape Interplay

Cancer cells accumulate metabolic alterations that allows access to conventional and unconventional nutrient sources for biomass formation, thus sustaining deregulated proliferation. Additionally, selected metabolites affect cancer cells’ fate, as well as, neighboring normal cells. The deregulated uptake of glucose and glutamine, as well as the capacity for nutrient acquisition under unfavorable conditions constitute two hallmarks of cancer metabolism (Pavlova and Thompson, 2016). These lead to intracellular metabolic reprogramming fostering the use of glycolytic and TCA cycle intermediates for biosynthesis and NADPH production and to increased nitrogen demand for nucleotide biosynthesis, the third and fourth hallmarks of cancer metabolism, respectively. Consequently, these metabolites lead to gene deregulation in cancer cells and also in tumor microenvironment (Katada et al., 2012).

**FIGURE 4:**
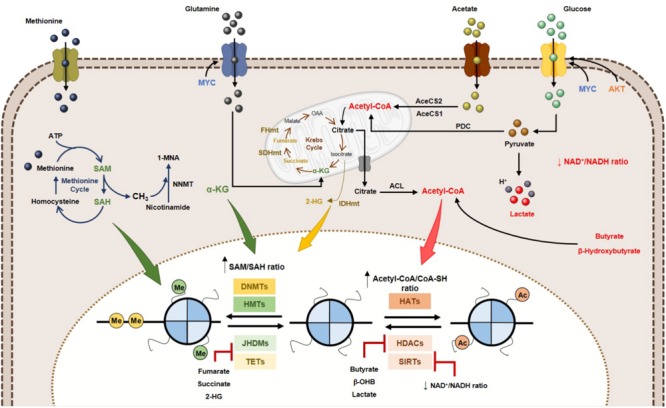
Effect of metabolites on DNA and histone code in human cancer. Tumor metabolome modulates several epigenetic mechanisms, thus contributing to gene expression deregulation. Signaling pathways involving myc and HIF-1α transcription factors upregulate the glucose and glutamine metabolism, which contribute to increased acetyl-CoA and α-KG production. High acetyl-CoA: CoA-SH ratio favoring HATs activity and consequently increased acetylation profile of tumor cells. Lactate, butyrate and β-hydroxybutyrate have been described as endogenous HDAC inhibitors. NAD^+^: NADH ratio is involved in Sirtuins’ activity regulation. SAM, synthetized from the essential amino acid methionine, is the substrate for HMTs and DNMTs. Other metabolites, such as fumarate, succinate and 2-HG has been identified as KDMs and TETs inhibitors, due to a structurally similarity to α-KG metabolite, a co-substrate to these enzymes. 2-HG, 2-hydroxyglutarate; AceCS1, acyl-CoA synthetase short-chain family member 1; AceCS2, acyl-CoA synthetase shirt-chain family member 2; ACL, ATP-citrate lyase; DNMTs, DNA methyltransferases; FHmut, fumarate hydratase mutant; HATs, histone acetyltransferases; HDACs, histone deacetylases; HMTs, histone methyltransferases; IDHmut, isocitrate dehydrogenase mutant; JHDMs, Jumonji-C domain-containing histone demethylases; PDC, pyruvate dehydrogenase complex; SAH, *S*-adenosylhomocysteine; SAM, *S*-adenosylmethionine, SDHmut; SIRTs, sirtuin’s; TETs, ten-eleven translocation family; α-KG, alpha-ketoglutarate.

Many of the chemical modifications in DNA and histones derive from intermediates of cellular metabolic pathways. This indicates that fluctuations in metabolic levels influence the deposition and removal of chromatin modifications (Schvartzman et al., 2018). The relationship between epigenetic regulation and metabolism is, however, complex. Metabolic reprogramming in cancer cells is considered one of the non-genetic factors to alter epigenetic landscape (Kim and Yeom, 2018). Cellular metabolism and epigenome interact with each other and with genetic and molecular drivers of cancer, bidirectionally (Kinnaird et al., 2016). A key characteristic defining the crosstalk between metabolism and chromatin is dependence of kinetic and thermodynamic properties of these interactions with dynamic range of physiological concentrations for corresponding metabolites (Kaelin and McKnight, 2013; Etchegaray and Mostoslavsky, 2016). Metabolites involved in this network include SAM, acetyl-CoA, NAD^+^, α-KG, among others (Figure 4). Additionally, metabolites such as *S*-adenosylhomocysteine (SAH), CoA, β-hydroxybutyrate, fumarate, succinate, lactate and 2-hydroxyglutarate (2-HG) modify enzyme activity, often by competitively inhibiting substrate utilization (Figure 4) (Reid et al., 2017). The role of the abovementioned metabolites in epigenetic regulation will be described in the following section: “Metabolites and DNA/Histone Methylation,” “Metabolites and Histone Acetylation,” and also in “Oncometabolites and Epigenetic Regulation.” The importance of metabolite pools in epigenetic landscape will be addressed in Section “Metabolites Pools in Subcellular Compartments.”

### Metabolites and DNA/Histone Methylation

DNA methylation is the most extensively studied epigenetic alteration in cancer, which typically affects promoter regions of cancer-related genes, leading to transcriptional repression (Kulis and Esteller, 2010). Unlike acetylation, histone methylation does not affect chromatin ionic charge, but functions as docking site for recruiting specific proteins/transcription factors. Histone methylation is an epigenetic mark associated with transcriptional repression or activation depending on the type of residue and the number of methyl group (Greer and Shi, 2012). In both cases, activity is dependent of *S*-adenosyl-methionine (SAM), a methyl donor product of serine-glycine one-carbon metabolism and methionine cycle, which is synthetized from ATP and methionine by the enzyme methionine adenosyltransferase (MAT) (Figure 4). SAM provides methyl groups which consistently release *S*-adenosyl-homocysteine (SAH), a potent inhibitor of DNMTs and HMTs (Mentch et al., 2015; Wong et al., 2017). Thus, SAM/SAH cellular ratio is a major determinant of chromatin methylation (Figure 4). In fact, increased SAM/SAH ratio associates with tumour suppressor genes’ hypermethylation and inappropriate silencing, whereas decreased SAM/SAH ratio contributes to reduced methylation at oncogenes’ promoters (Wong et al., 2017).

Glycine-*N*-methyltransferase (GNMT) is involved in SAM levels’ homeostasis. Indeed, GNMT deficiency was associated with RASSF1 and SOCS2 promoter methylation, and oncogenic pathways activation in hepatocellular carcinoma (Mudd et al., 2001; Martinez-Chantar et al., 2008). Additionally, aberrant expression of Nicotinamide *N*-methyltransferases (NMMT, a limiting enzyme that metabolizes SAM) has been observed in lung, liver, kidney, bladder and colon cancers (Wu et al., 2008; Tang et al., 2011; Thomas et al., 2013; Zhang et al., 2014). Cell lines overexpressing NMMT display significantly decreased HMTs activity and, consequently, histone methylation marks, especially at H3K4, H3K9, H3K27 and H4K20, associating with more aggressive/pluripotent phenotype. Conversely, because DNMTs have lower Km values for SAM compared to HMTs, DNA methylation is not affected by aberrant NMMT expression levels (Wong et al., 2017). Moreover, amino acid transporters overexpression by cancer cells may directly increase methionine uptake (Fuchs and Bode, 2005; Haase et al., 2007). Likewise, serine is also in high demand by cancer cells, contributing to increased ATP availability in cancers cells and provision of SAM, which is synthesized from methionine (Rabhi et al., 2017). Interestingly, increased methylthioadenosine (MTA) concentration in cancer cells harbouring 5-methylthioadenosine phosphorylase (MTAP) deletions results in decreased H4R3me2 mark and, consequently, arginine methyltransferase 5 (PRMT5) inhibition (Kryukov et al., 2016).

DNA and histone methylation is also regulated by DNA and histone demethylases. TET proteins catalyse 5-methyl-cytosine (5-mC) oxidation, generating 5-hydroxymethyl-cytosine (5-hmC), allowing for demethylation of aberrantly methylated cytosine residues (Huang and Rao, 2014). Histone demethylases dependent of flavin (LSD1) and Jumonji C-domain-containing (JMJD) enzymes demethylate lysine marks (Dimitrova et al., 2015). Metabolites may serve as substrate and/or co-factors for DNA and histone demethylases. TCA cycle generates several intermediary metabolites, some of which involved in DNA/histone demethylases activity. Concerning histone demethylation reaction catalysed by LSD1, it is accomplished by reduction of co-factor FAD^+^ to FADH_2_ at mitochondrial level. LSD1 demethylase activity appears to control metabolism favouring de novo fatty acids synthesis over gluconeogenesis in hepatocytes and adipocytes (Zheng et al., 2015). In tumour cells, LSD1 overexpression leads to methyl group removal from H3K9me and H3K4me, favouring tumour progression, cell proliferation and stemness (Hino et al., 2016).

Both JMJDs and TETs are dioxygenases dependent of α-ketoglutarate (α-KG) as co-factor (Figure 4), being inhibited by TCA cycle intermediates succinate and fumarate (Xiao et al., 2012): α-KG is produced from isocitrate by mitochondrial enzymes isocitrate dehydrogenase 2 (IDH2) and 3 (IDH3) as an intermediary of TCA cycle. In addition to isocitrate, α-KG is also synthetized from amino acids such as arginine, glutamine, histidine and proline (Figure 4) (Etchegaray and Mostoslavsky, 2016; Rabhi et al., 2017; Kim and Yeom, 2018). The α-KG/succinate ratio regulates chromatin status in embryonic stem cells (ESCs) through JMJD3 and Tet1/Tet2 demethylation of H3K9me3, H3K27me3 and H4K20me (Carey et al., 2015). Although Jmj-KDMs expression deregulation has been reported in various cancers, how fluctuations in α-KG correlate with Jmj-KDM-driven cancers is not completely understood. In melanoma cells, glutamine depletion at hypoxic tumour core associates with histone hypermethylation, mostly H3K27 methylation, due to reduced α-KG. H3K27 methylation is particularly increased in genes associated with cancer cells dedifferentiation and confers resistance to BRAF^V 600E^ inhibitors (Pan et al., 2016). Finally, α-KG is an important metabolite for activity of other dioxygenases, including RNA N^6^-methyladenosine (m^6^A) demethylation and EglN prolyl-4-hydroxylation (Schvartzman et al., 2018).

Overall, tumor cells modulate the expression of several metabolic enzymes necessary for the maintenance of SAM and α-KG levels, enabling an epigenetic profile favorable to less differentiation and higher proliferative capacity.

### Metabolites and Histone Acetylation

Histone acetylation, catalyzed by HATs, transfer an acetyl group from acetyl-CoA to the amino group of specific histone lysine residues, allowing transcriptional access to DNA by positive charge neutralization (Verdin and Ott, 2015). This contributes to open chromatin conformation and recruitment of several transcriptional activators, entailing transcriptional activation.

Acetyl-CoA is a central metabolite coordinating the activity of HATs, since increased levels contribute to increased histone acetylation (Figure 4) (Lee and Workman, 2007). This metabolite is a key intermediary produced during catabolism and anabolism, both in mitochondria and cytoplasm, associating with breakdown of carbohydrates and fats, via glycolysis and β-oxidation, respectively (Pietrocola et al., 2015). Additionally, acetyl-CoA might derive from ketone bodies and amino acids (Pietrocola et al., 2015). In mitochondria, pyruvate generated from glycolysis and β-oxidation is converted to acetyl-CoA. As a mitochondrial impermeable metabolite, citrate produced in Krebs cycle from acetyl-CoA is transported to cytoplasm and subsequently converted to acetyl-CoA through ATP-citrate lyase (ACL) (Figure 4) (Wellen et al., 2009; Zaidi et al., 2012; Kim and Yeom, 2018). Additionally, when glucose availability is limited and/or in hypoxia conditions, acetate may be a source of acetyl-CoA. Acetate that enters the mitochondria is used to acetyl-CoA synthesis through mitochondrial acetyl-CoA synthetase 2 (AceCS2) or promotes acetyl-CoA production in cytoplasm through acetyl-CoA synthetase 2 (AceCS1) (Figure 4) (Kim and Yeom, 2018). Acetyl-CoA levels are quite dynamic and directly dependent of nutrient availability. Indeed, histone acetylation is regulated by acetyl-CoA absolute levels and the ratio acetyl-CoA/coenzyme A in cancer cells (Lee et al., 2014). The expression of ACL and the availability of citrate modulate cellular acetyl-CoA levels. In colorectal cancer, ACL silencing suppressed histone acetylation (Wellen et al., 2009) whereas ACL overexpression was reported in different tumours (Migita et al., 2008), probably contributing for nuclear acetyl-CoA pool, necessary for histone acetylation and glycolytic enzymes expression.

The glycolytic flux and mitochondrial citrate production, subsequently migrating to cytosol and nucleus is promoted by metabolic reprogramming in cancer cells. In pancreatic adenocarcinoma, Akt signalling activation, through activated KRAS^G12D^, promotes nuclear acetyl-CoA accumulation and ACL phosphorylation, inducing histone acetylation (Lee et al., 2014). Additionally, MYC increases mitochondrial export of acetyl-groups and upregulates HAT-GCN5 expression, inducing H4 acetylation (Knoepfler et al., 2006). After glucose, glutamine is the main acetyl-CoA source in tumour cells. In glucose deprivation, glutamine is used as substrate and acetyl-CoA production in TCA cycle favours histone acetylation, stimulating tumour cell proliferation and growth (Le et al., 2012; Lu and Thompson, 2012; McDonnell et al., 2016; Rabhi et al., 2017).

Regardless of Warburg effect and hypoxia occurring in solid tumors, increased glucose to lactate conversion has been observed. This switch is enhanced by HIF-1α, a glycolytic metabolism activator that induces PDK, a TCA cycle suppressor. Under these conditions, tumor cells often use acetate as alternative carbon source for acetyl-CoA production by AceCS2 and although tumor cells display low acetate concentration, this metabolite may be advantageous (Comerford et al., 2014). Higher acetate consumption constitutes a tumor cells’ adaptation to increased acetate secretion by surrounding stromal cells, which results from acidification due to lactate production (van der Knaap and Verrijzer, 2016). Moreover, increased cellular pH has been associated with increased histone acetylation and reduced acetate and protons co-exportation (McBrian et al., 2013). These observations suggest that chromatin might act as sensor of carbon flux and cellular pH, thus being implicated in cellular physiology regulation. In this context, acetyl-CoA acts as a metabolic biosensor that triggers upregulation of specific genes involved in growth and proliferation in response to nutrient availability through histone acetylation.

The antagonistic functions of HDACs and HATs regulate histone acetylation. Lysine/histone deacetylases (KDAC/HDAC) catalyse removal of the acetyl group from lysine residues of histones, favouring condensed chromatin status and consequent gene transcriptional repression (Yoshida et al., 2017). HDACs are divided in four classes according to structural and mechanistic similarities: zinc-dependent class II, II and IV (classical HDACs) and NAD^+^ dependent class III (sirtuins’ family) (Seto and Yoshida, 2014).

Deacetylation reactions are also metabolic responsive. In addition to the well-known HDAC inhibitors (class I, II and IV), trichostatin (TSA) and suberoylanilide hydroxamic acid (SAHA), HDAC activity can be antagonized by different cellular metabolites (Figure 4) (Marchion and Munster, 2007). Butyrate, a short fatty acid, used as energy source for colon cell growth, inhibits class I, II and IV HDAC activity (Candido et al., 1978; Fan et al., 2015). Additionally, in breast cancer cell lines, ketogenic bodies, namely β-hydroxybutyrate, were shown to reduce the activity of class I and II HDACs (Martinez-Outschoorn et al., 2012). Furthermore, lactate has been shown to inhibit HDAC activity in cancer cells, similar to TSA and butyrate (Latham et al., 2012; Wagner et al., 2015). In cancer cells, histone deacetylation mediated by HDACs causes tumour suppressor genes silencing (Nakagawa et al., 2007). Metabolic reprogramming can affect histone acetylation by accumulation of metabolites that inhibit histone deacetylases. In colon cancer cells, Warburg effect leads to accumulation of butyrate due to suppression to acetyl-CoA conversion. Consequently, increased butyrate inhibits HDAC activity, upregulating pro-apoptotic genes. When glycolytic metabolism is inhibited, butyrate promotes acetyl-CoA production facilitating colon cancer cell growth. Thus, metabolic reprogramming can instruct cancer cells to distinctively utilize metabolites to mediate differential epigenetic modifications (Donohoe et al., 2012). Remarkably, a similar effect was also observed with β-hydroxybutyrate in tumour brain cells. This metabolite is produced from ketogenesis and used as energy source by normal brain cells. Upregulated glycolytic rates suppress conversion in tumour cells, resulting in β-hydroxybutyrate accumulation which inhibits histone deacetylation (Newman and Verdin, 2014). Furthermore, in tumour cells, enhanced glycolytic phenotype increases lactate production that is exported to tumour microenvironment. This cellular lactate may negatively regulate HDAC activity and, consequently, gene expression. Interestingly, in breast cancer cells, lactate induced a distinctive gene expression signature related with stemness (Martinez-Outschoorn et al., 2011). Thus, HDAC inhibition by lactate might be involved in cancer cell fate decision.

NAD^+^ is an important cofactor for histone deacetylases class III (sirtuins) activity. This important redox co-factor is required by many enzymes involved in catabolic or oxidative pathways including glycolysis, TCA cycle and β-oxidation of fatty acids (Figure 4) (Imai and Guarente, 2014). NAD^+^ levels determines sirtuins activity, depending on nutrient availability. When energy is in excess, NAD^+^ is depleted, generating lower NAD^+^/NADH ratio, inhibiting sirtuins’ activity (Li and Kazgan, 2011; Canto et al., 2015). In contrast, NAD^+^ levels rise in energy deficiency situations, like physical exercise or caloric restriction (increased NAD^+^/NADH ratio leading to AMPK activation), entailing sirtuins’ activation (Canto et al., 2009). SIRT1 and SIRT6 are overexpressed in those conditions contributing to decreased histone (H3K9ac and H3K14ac) acetylation (Etchegaray and Mostoslavsky, 2016). In parallel, decreased glycolytic gene expression and increased gluconeogenesis gene expression also occurs, promoting cell survival (German and Haigis, 2015). Cancer cells rely on glycolysis even in the presence of oxygen, leading to low NAD^+^/NADH ratio and consequent inhibitory effect on sirtuins’ activity. Moreover, deviant gene transcription due to increased histone acetylation is caused by augmented activity of HATs (acetyl-CoA induced) and sirtuins, favouring tumour growth and progression (Wong et al., 2017). Indeed, SIRT6 was reported as tumour suppressor in pancreatic cancer (Kugel et al., 2016), as well as other isoforms in different tumours. Furthermore, in colorectal cancer, SIRT6 expression loss associated with glycolytic genes upregulation, promoting cellular transformation, tumour growth and aggressiveness (Sebastian et al., 2012). Thus, sirtuins may suppress tumorigenesis through epigenetic mechanisms that modulate metabolic reprogramming.

Cancer metabolic reprogramming has an important impact in the epigenetic machinery. Tumor cells with metabolic adaptation make use of glucose oxidation pathway, glutaminolysis and acetate, depending on the nutrient availability, enriching the nuclear acetyl-CoA pool for histone acetylation. The characteristic Warburg effect in tumor cells allows them to use the lactate and glycolytic metabolites, as HDAC inhibitors favor a hyperacetylated status which triggers upregulation of genes involved in cell proliferation.

### Oncometabolites and Epigenetic Regulation

Some metabolites are able to promote tumorigenesis by altering the epigenome, being defined as oncometabolites (Nowicki and Gottlieb, 2015). These oncometabolites, namely fumarate, succinate and 2-hydroglutarate, are generated in excess due to mutations in TCA cycle-associated enzymes. Mutations in genes encoding metabolic enzymes result in pathological accumulation of metabolites that may affect histone and DNA methylation. IDH1 and IDH2 mutations have been identified in acute myelogenous leukemia, lymphoma, glioblastoma, chondrosarcoma and other solid tumours (Yan et al., 2009; Ward et al., 2010; Cairns et al., 2012; Cancer Genome and Atlas Network, 2012). These loss-of-function mutations in IDH1/2 prevent conversion of α-KG to isocitrate, favouring synthesis of 2-hydroxyglutarate (2-HG), instead (Figure 4) (Dang et al., 2009). This oncometabolite is competitive inhibitor of α-KG, inhibiting TET and JmjC activity (Figure 4) (Xu et al., 2011). Moreover, 2-HG is also increased in breast (Terunuma et al., 2014) and renal cancer (Shim et al., 2014). 2-HG is the product of malate dehydrogenase 1 and 2, and LDHA, which has been linked with deficiency of L-2-hydroxyglutarate dehydrogenase and activation of MYC, in renal and breast cancer, respectively. Interestingly, the enantiomer S-2-HG is produced by LDHA under hypoxic conditions, also affecting histone methylation and hypoxic transcriptional responses (Intlekofer et al., 2015). *In vitro* enzymatic assays showed that 2-HG inhibits Tet1/2 activity, abrogating 5hmC formation in human cell lines (Figueroa et al., 2010). Additionally, IDH R132H mutant cells display CpG island methylator phenotype, similarly to gliomas and acute myeloid leukemia, with reduced Tet1/2 activity (Figueroa et al., 2010; Turcan et al., 2012).

High 2-HG levels in IDH mutants also have an impact on KDMs activity. Specifically, increased 2-HG levels preferentially inhibits KDM4A, KDM4C and KDM2A (Chowdhury et al., 2011). In human glioblastoma cell line, U87-MG, 2-HG increases H3K9me2, H3K27me2 and H3K79me2, as well as H3K4me3 and associates with pluripotent genes’ expression, hindering differentiation (Xu et al., 2011). Human IDH mutant gliomas display higher H3K79me3 levels than wild-type IDH (Lu et al., 2012). The limiting availability of α-K due to the production of 2-HG, in IDH mutants, suggest a metabolic interplay between TET and JmjC domain-dependent epigenetic dynamics.

Fumarate hydratase (FH) and succinate dehydrogenase (SDH) mutations were identified in several sporadic and hereditary cancers, causing accumulation of their substrates (Gaude and Frezza, 2014). High fumarate and succinate levels are α-KG competitive antagonists (Figure 4) (Xiao et al., 2012). FH and SDH mutants exhibit a methylator phenotype, with increased 5mC/5hmC ratio and H3K9 and H3K27 methylation, due to TETs and KDM2A/KDM4C inhibition, respectively, associating with downregulated transcriptional program, promoting metastasis and leading to increased invasiveness (Wong et al., 2017). In fact, gastrointestinal stroma tumors (GISTs), paraganglioma and pheochromocytoma harboring SDH mutations display genomic DNA hypermethylation (Killian et al., 2013; Letouze et al., 2013). Moreover, paraganglioma patients with SDH and FH deficiency associated with DNA CpG island methylator phenotype have worse prognosis compared with other subtypes (Letouze et al., 2013). Thus, FH and SDH genetic mutations can lead to fumarate and succinate accumulation, inducing tumorigenesis via epigenetic deregulation.

A recent report demonstrated that FH loss-of-function mutation and subsequent accumulation of fumarate promotes epithelial-mesenchymal transition (EMT) through fumarate-dependent inhibition of TET demethylases and subsequent induction of genes necessary for EMT (Sciacovelli et al., 2016). Moreover, high succinate levels may inhibit EglN prolyl-4-hyroxylases by HIF-1α and HIF-2α stabilization, in FH and SDH mutant tumors (Koivunen et al., 2012). Interestingly, 2-HG may also have a tumor suppressive effect in non-mutant IDH leukemias by inhibiting m6A demethylase and destabilizing MYC transcripts (Su et al., 2018).

Genetic insults associated with metabolic enzyme mutations contribute to oncometabolites production, in which, themselves, are capable to induce tumorigenesis via epigenetic deregulation.

### Metabolite Pools in Subcellular Compartments

Currently, it is acknowledged that metabolic enzymes that modulate epigenetic landscape disclose nuclear localization, specifically ACL, AceCS1 and AceCS2, in glioma and colon cancer cell lines (Wellen et al., 2009). Nonetheless, acetyl-CoA resides in different cellular components: mitochondrial, cytosolic and nuclear. Mitochondrial acetyl-CoA is key for TCA cycle and mitochondrial ATP production, whereas cytosolic acetyl-CoA pool supplies fatty acids, cholesterol and hexosamine biosynthesis pathways. Acetyl-CoA derived from glucose oxidation and fatty acids β-oxidation generates citrate inside mitochondria (Kinnaird et al., 2016). ACL and AceCS2 enzymes that can be found in nucleus and cytoplasm, participate in overall histone acetylation regulation (Takahashi et al., 2006; Wellen et al., 2009). AceCS2 is predominantly expressed in nuclei of tumor cells (Comerford et al., 2014), particularly under oxygen and/or glucose limited condition, as well as acetate (Sivanand et al., 2018). Furthermore, nuclear acetate might be also released from chromatin by HDACs and recruited by AceCS2 to supply acetyl-CoA production for histone acetylation (Sivanand et al., 2018). Likewise, AceCS2 nuclear localization has been associated with transcriptional activation of autophagy and lysosomal biogenesis genes. Similarly, nuclear ACL generates acetyl-CoA pools upon DNA damage, facilitating histone acetylation required for efficient double-strand break repair (Sivanand et al., 2017). Furthermore, pyruvate dehydrogenase complex (PDC) translocate from mitochondria to nucleus where it may generate local high acetyl- CoA concentrations to fuel histone acetylation required for gene transcription involved in S-phase of cell cycle (Sutendra et al., 2014) and lipid biogenesis (Chen et al., 2018). Nuclear localization of acetyl-CoA synthesizers in some biological conditions, including cancer, suggest that acetyl-CoA production is spatially regulated and that acetylation status controls metabolism. The ACL and AceCS2 enzymes promote acetyl-CoA production at nuclear level.

## Crosstalk Between Metabolites and Epigenetics

Currently, three different mechanisms support the relevance of molecular and metabolic rewiring in the epigenetic landscape and how these epigenetic modifications influence cancer biology (Kinnaird et al., 2016).

**FIGURE 5:**
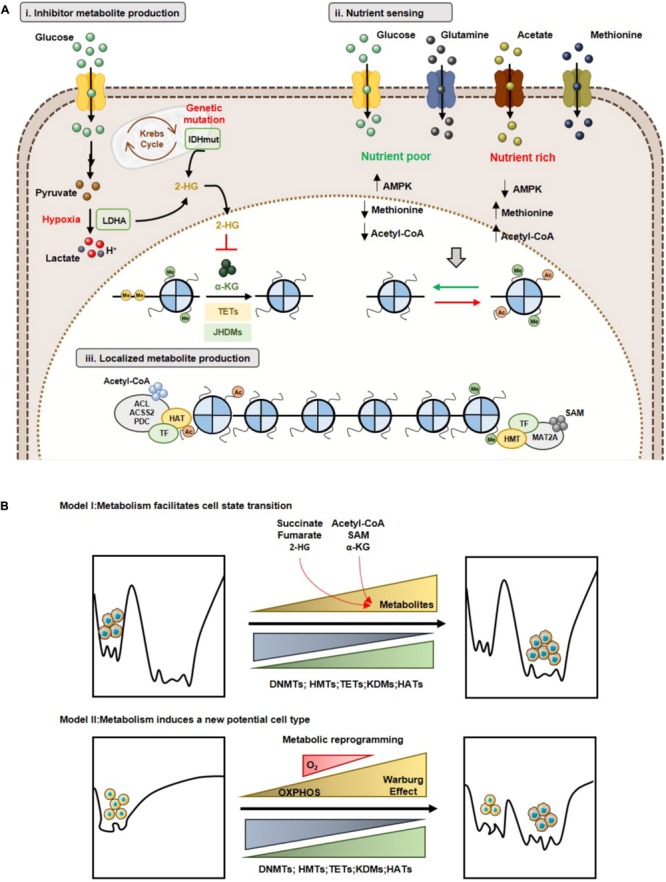
Metabolism and epigenetic landscape interplay. **(A)** Models of cooperation between metabolism and epigenome: **(i)** Inhibitor metabolite production and chromatin regulation; **(ii)** Nutrient sensing and chromatin regulation; **(iii)** Localized metabolite production and chromatin regulation. **(B)** Metabolic reprogramming and Waddington’s epigenomic landscape. Model I: Changes in metabolite levels may lead to reorganization of specific chromatin marks without affecting the shape of epigenomic landscape (Top). Model II: Metabolic reprogramming leads to a new cell state with a different epigenomic landscape (Bottom).

The first model concerns inhibitory metabolites produced by tumor cells, that affect usage of α-KG by TETs and KDMs (Kinnaird et al., 2016). As previously stated (see Oncometabolites and Epigenetic Regulation), several oncometabolites are produced by mutations in genes encoding for metabolic enzymes, which lead to increased histone/DNA aberrant methylation, impacting on cancer gene expression profiles (Figure 5Ai). The second model, concerns the nutrient sensing capacity and its effect in chromatin regulation (Figure 5Aii) (Kinnaird et al., 2016). A canonical example of metabolite sensing is AMPK activation in low nutrient conditions (Hardie et al., 2012). Additionally, SAM pool dependence on diet-derived methionine also illustrates the relevance of this concept (Lim and Song, 2012). In fact, it is acknowledged that SAM serum levels, as well as methylation levels, vary depending on cancer patients’ diet (Schernhammer et al., 2010). Finally, acetyl-CoA levels sensing optimizes the metabolic needs of growth and proliferation (Cai et al., 2011; Donohoe et al., 2012). The higher tumor heterogeneity is reflected in different metabolic behaviors. Thus, cells might present a specific metabolic phenotype, depending on the nutrient availability. Consequently, tumor cells display diverse metabolite sensors, which differential impact on epigenetic landscape. Moreover, metabolic cooperation among tumor cells, especially metabolites sensors availability, regulates histone writers’ expression favoring a proliferative profile. The third model, involves localized metabolite production and chromatin regulation (Kinnaird et al., 2016). Indeed, direct metabolic enzymes’ recruitment to specific chromatin sites facilitates site-specific cofactors or substrate production and consequent histone modifications. Thus, SAM synthase isoform type 2 (MAT2A) is required for histone methylation at specific sites and the same occurs for ACL and PDC that locally generate acetyl-CoA to be used by specific HATs to acetylate histones (Figure 5Aiii). Hence, metabolic influence on the cancer epigenome may occur through multiple mechanisms and, importantly, these are not mutually exclusive, as tumors probably undertake all three modes of regulation depending of the tumor microenvironment context.

Concerning the impact of metabolic-epigenetic crosstalk, two models are currently accepted in line with Waddington’s landscape context (Feinberg, 2007) and described recently by Reid MA et al. (Reid et al., 2017). Accordingly, model I suggests that metabolic reprogramming facilitates transition from one cell type to another, by changing specific chromatin modulators. Indeed, changes in metabolite levels (e.g., α-KG, methionine) modulate both the activity of DNMTs/KDMs, promoting specific epigenetic marks’ reorganization, facilitating cell differentiation (Reid et al., 2017). Cell transition might be due to nutrient availability in tumor microenvironment that promotes metabolic reprogramming and, consequently, a specific metabolite sensor activity. This metabolic sensor activity might alter acetylation cells’ methylation profile, which allows for cell transition, as described in model I (Figure 5B). Model II, sustains that metabolic reprogramming changes in Waddington’s landscape induces the formation of a new stable epigenetic phenotype. Thus, alterations in cellular metabolism may induce gene expression reprogramming associated with chromatin remodeling or may directly influence the availability of substrates and cofactors of chromatin-modifying enzymes’ (Reid et al., 2017). In this case, metabolic reprogramming induced by hypoxia might explain the transition to a new cell type, as referred in model II. During hypoxia development, shift from TCA cycle to Warburg effect is observed. This metabolic reprogramming could lead to chromatin regulators’ upregulation or changes in availability of substrates and cofactors, generating a cell pool with a new phenotype, entailing an aggressive and/or resistant phenotype (Figure 5B).

Despite evidence that cellular metabolic status affects epigenetic landscape regulation, it might be hypothesized whether metabolism rheostat promotes cell proliferation, cell death or differentiation. Indeed, high nuclear acetyl-CoA/CoA-SH ratio promotes acetylation of histone and transcription factors involved in cell cycle progression and proliferation (Cai et al., 2011; Lee et al., 2014). Additionally, cell differentiation, survival and death are affected by acetyl-CoA availability, particularly acetyl-CoA/CoA-SH ratio (Eisenberg et al., 2014; Moussaieff et al., 2015). Regarding global methylation, α-KG and oncometabolites production are key for chromatin organization and differentiation regulation. In ESCs, increased α-KG/succinate ratio decreases suppressive methylation marks on DNA and histones, promoting pluripotency (Carey et al., 2015). Nevertheless, DNA and histone demethylases inhibition by succinate, fumarate and 2-HG production promotes proliferation over differentiation of tumor cells (Figueroa et al., 2010; Lu et al., 2012). Finally, acetylation may directly regulate function or intracellular localization of several proteins implicated in carcinogenesis. Indeed, acetylation promotes metabolic rewiring by directly suppressing mitochondrial activity, increasing glycolysis, associating with proliferative phenotype (Zhao et al., 2014). Hence, the identification of nuclear and cytoplasmic proteins which are acetylated in an acetyl-CoA dependent manner is mandatory to fully understand how cellular and molecular events are affected by nutrient availability, opening new therapeutic opportunities for cancer treatment.

## Epigenetic-Metabolism Crosstalk in Cancer Cells as a Therapeutic Target

**FIGURE 6:**
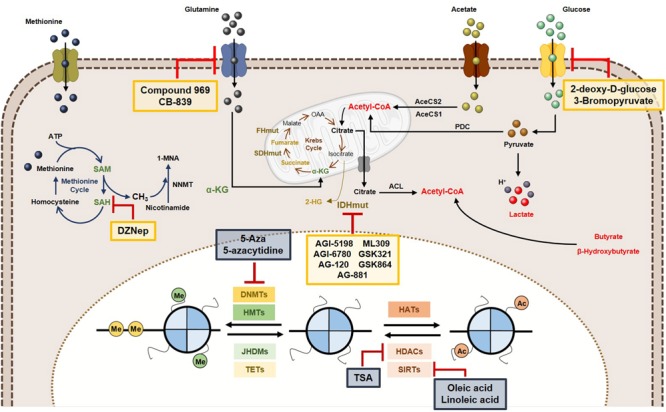
Metabolic and epigenetic targeting inhibitors. Yellow box represents the inhibitors targeting tumor cell metabolism. Blue box are representative of epigenetic enzymes inhibitors. Abbreviations: 2-HG, 2-hydroxyglutarate; AceCS1, acyl-CoA synthetase short-chain family member 1; AceCS2, acyl-CoA synthetase shirt-chain family member 2; ACL, ATP-citrate lyase; DNMTs, DNA methyltransferases; FHmut, fumarate hydratase mutant; HATs, histone acetyltransferases; HDACs, histone deacetylases; HMTs, histone methyltransferases; IDHmut, isocitrate dehydrogenase mutant; JHDMs, Jumonji-C domain-containing histone demethylases; PDC, pyruvate dehydrogenase complex; SAH, *S*-adenosylhomocysteine; SAM, *S*-adenosylmethionine, SDHmut; SIRTs, sirtuin’s; TETs, ten-eleven translocation family; α-KG, alpha-ketoglutarate.

Considering the complex relationships between epigenetics and metabolism, some innovative cancer therapies have been suggested, as targeting tumor metabolism might reverse epigenetic dysregulation and epigenetic-modifying drugs may modulate cancer metabolism (Figure 6).

### Tumor Metabolism Inhibitors

In cancer cells, increased histone acetylation is, in part, caused by the elevated glycolytic flow (and associated flux of glucose), mediated by acetyl-CoA and citrate. Thus, glycolysis inhibition may lead to histone acetylation modulation. 2-Deoxyglucose (2-DG), a glucose analog may competitively inhibit G6P production, hindering the glycolytic pathway (Chen and Gueron, 1992). Furthermore, 2-DG treatment suppresses acetyl-CoA levels, leading to global histone H3 and H4 decrease in several cancer cell lines and associates with compromised DNA repair and cancer cells sensitization to DNA-damaging agents (Liu et al., 2015). Another glycolysis inhibitor, 3-bromopyruvate, decreases acetyl-CoA and induces differentiation in embryonic stem cells (Moussaieff et al., 2015).

Several inhibitors targeting glutaminase (GLS) (which deaminates glutamine to glutamate) have been developed. Compounds 968 and CB-839 are two GLS inhibitors. In breast cancer cells, decreased expression of several cancer-associated genes was observed as a result of alterations in H3K4 methylation and H4K16 acetylation due to 968 (Simpson et al., 2012), whereas CB-839 is currently in Phase I trial in solid and hematological cancers (Robinson et al., 2007; Wang et al., 2010).

IDH mutations are key events in epigenetic landscape of leukemias and gliomas. IDH1/2 inhibition has been suggested to suppress 2-HG production. In mutant IDH glioma cells, AGI-5198 was shown to inhibit 2-HG production and cell growth, inducing H3K9me3 and H3K27me3 demethylation, not affecting DNA methylation (Rohle et al., 2013). The same was reported in human IDH mutant chondrosarcoma cells (Li et al., 2015). Subsequently, novel mutant IDH1^R132H^ inhibitors, including AG-120, AG-881, ML309, GSK321 and GSK864 have shown efficacy (Wong et al., 2017). Additionally, AG-221, a first-in-class inhibitor of mutant IDH2, leads to 2HG reduction in IDH2 mutant leukemia and survival benefit in primary human IDH2 mutant AML xenografts (Yen et al., 2017). This IDH2 inhibitor underwent Phase I and Phase II clinical trials, in which effective 2HG levels decrease was observed both in bone marrow and in plasma, achieving sustainable remission of disease in some patients with advanced hematologic malignancies harbouring IDH2 mutations (Wong et al., 2017). Likewise, AGI-6780, another mutant IDH2 inhibitor, caused demethylation of DNA and histones, reversing gene expression patterns that were acquired during tumorigenesis owing to epigenetic deregulation (Wang et al., 2013).

Because SAM availability is critical for DNMTs and HMTs activity and SAH hydrolase is essential for methylation homeostasis maintenance, SAH hydrolase inhibitors have emerged, namely DZNep (3-deazaneplanocin A) (Glazer et al., 1986). DZNep was ineffective in reactivating silenced genes due to promoter methylation in cancer cells, although it globally inhibited DNA and histone methylation, reactivating a subset of developmental genes. However, a synergistic effect against leukemic cells was observed when combined with the DNMT inhibitor 5-aza-2′-deoxycytidine (5-Aza), through activation of genes silenced by histone and DNA methylation (Momparler et al., 2014; Momparler and Cote, 2015).

### Epigenetic Enzymes’ Inhibitors

Inhibition of DNMTs effectively reverses DNA methylation and two inhibitors (5-Aza and 5-azacytidine) were approved by the American and European regulatory agencies for treatment of selected hematological maligancies. In solid tumors, results from clinical trials were less effective and the effect of these inhibitors on cancer metabolism is currently unknown. Nevertheless, in IDH mutant cancers inhibitors of DNMTs were able to reverse DNA methylation. Treatment of IDH1 mutant glioma cells suppressed tumour growth and was effective in inducing differentiation compared to mutant IDH inhibitors (Borodovsky et al., 2013; Turcan et al., 2013).

Furthermore, evidence that inhibition of HDAC affects the metabolism of cancer cells is growing. Colorectal cancer cell line HT29 treated with a combination of butyrate and TSA (both HDAC inhibitors) disclosed reduced glycolytic metabolism (Alcarraz-Vizan et al., 2010). In a different tumour model (multiple myeloma), the HDAC inhibitors vorinostat and valproate treatment effectively abrogated the expression of GLUT1 and HKI activity (Wardell et al., 2009). Exposure of H460 lung cancer cell line to butyrate and TSA resulted in a reversal of the glycolytic phenotype, with transition to dependency from oxidative phosphorylation (Amoedo et al., 2011) and a similar effect was disclosed in breast cancer cells (Rodrigues et al., 2015). Thus, effective inhibition of HDAC activity may reverse aerobic glycolysis in cancer. Because sirtuin family members, play an important role in metabolic regulation of cancer cells, especially SIRT6, specific inhibitors may provide an additional strategy to target cancer cell metabolism (Feldman et al., 2013).

## Conclusion

Altered metabolism and epigenetic deregulation have mutual influence in adaptation of cancer cells to a constantly changing environment. Metabolic rewiring in cancer cells affects the epigenome facilitating tumour development and progression. Specifically, acetyl-CoA pools are key in epigenetic control. Depending on metabolic pathway involved in acetyl-CoA production, histone acetylation patterns in different transcriptional gene targets may engage. Thus, the specificity of the metabolite-driven epigenetic regulation of targets is important to allow better understanding of cancer biology. Additionally, identification of transcription factors activated in different metabolic states, as well as the role of metabolic enzymes in nuclear compartment, will allow for discovery of mechanisms underlying integration of metabolic signalling in chromatin.

Most available data on epigenetic and metabolic crosstalk in cancer cells derives from 2D cell culture models, which do not realistically portray the complexity of this interaction *in vivo*, especially when the critical role of TME is considered. In fact, epigenetic drugs may have limited success in solid tumours with extensive hypoxic regions (Braiteh et al., 2008; Chu et al., 2013), which has been associated not only with tumour progression and aggressiveness but also with therapy resistance (Wilson and Hay, 2011). Moreover, hypoxic tumour cells display epigenetic abnormalities, namely DNA hypomethylation and histone hyperacetylation (Johnson et al., 2008). Thus, approaches that target epigenetic mechanisms should consider the impact of both tumour microenvironment and metabolism.

Previous studies have demonstrated that inhibition of epigenetic factors (e.g., HDACs, DNMTs) has an impact in cancer cell metabolism, although further studies are required to fully understand its effectiveness and the underlying mechanisms. Furthermore, clinical trials should incorporate biomarker analysis to unravel epigenomic and metabolomic markers allowing for identification of patient subsets that may benefit most from metabolic/epigenetic modulators treatment. Additionally, combining epigenetic and metabolic targeting might provide a more effective means of inhibiting tumour progression. Overall, in view of the tumour microenvironment’s key role in epigenetic plasticity, patients might also benefit from inclusion of other therapeutic strategies that target TME components (e.g., anti-angiogenics, immune checkpoint inhibitors), as well as conventional chemotherapy. Altogether and from a theoretical standpoint, these combinations are likely to positively impact on cancer patients’ management.

## Author Contributions

VM-G, AL, and CJ conceptualized the paper. VM-G and AL collected, analyzed the information, and elaborated the figures. VM-G and AL drafted the manuscript. RH and CJ revised the paper. All the authors read and approved the final manuscript.

## Conflict of Interest Statement

The authors declare that the research was conducted in the absence of any commercial or financial relationships that could be construed as a potential conflict of interest.
