# Corrigendum: Metabolism and Epigenetic Interplay in Cancer: Regulation and Putative Therapeutic Targets

**DOI:** 10.3389/fgene.2019.00784

**Published:** 2019-09-13

**Authors:** Vera Miranda-Gonçalves, Ana Lameirinhas, Rui Henrique, Carmen Jerónimo

**Affiliations:** ^1^Cancer Biology and Epigenetics Group, Research Center (CI-IPOP), Portuguese Oncology Institute of Porto, Porto, Portugal; ^2^Master in Oncology, Institute of Biomedical Sciences Abel Salazar (ICBAS), University of Porto, Porto, Portugal; ^3^Department of Pathology, Portuguese Oncology Institute of Porto, Porto, Portugal; ^4^Department of Pathology and Molecular Immunology, Institute of Biomedical Sciences Abel Salazar (ICBAS), University of Porto, Porto, Portugal

**Keywords:** cancer metabolism, metabolites, DNA methylation, histone modifications, epigenetic plasticity

In the original article there was partial textual overlap with a review by Wong et al. 2017 from which this article drew from and built substantially on. This correction has been made with these sentences replaced with those of the same meaning. The authors apologize for this error and state that this does not change the scientific conclusions of the article in any way. The original article has been updated. 

A correction has therefore been made to the *Abstract*:

“Alterations in the epigenome and metabolism affect molecular rewiring of cancer cells facilitating cancer development and progression. Modulation of histone and DNA modification enzymes occurs owing to metabolic reprogramming driven by oncogenes and expression of metabolism-associated genes is, in turn, epigenetically regulated, promoting the well-known metabolic reprogramming of cancer cells and, consequently, altering the metabolome. Thus, several malignant traits are supported by the interplay between metabolomics and epigenetics, promoting neoplastic transformation. In this review we emphasize the importance of tumour metabolites in the activity of most chromatin-modifying enzymes and implication in neoplastic transformation. Furthermore, candidate targets deriving from metabolism of cancer cells and altered epigenetic factors is emphasized, focusing on compounds that counteract the epigenomic-metabolic interplay in cancer.”

A correction has been made to ***Epigenetic Mechanisms in Cancer: a Brief Overview***, paragraph three:

“The most studied epigenetic alterations associated with neoplastic phenotype are variation in DNA methylation, alterations in histone proteins structure through post-translational modifications and histone variants (**Figure 1**) (Biswas and Rao, 2017). Additionally, microRNAs, which are small RNA molecules (22 nucleotides long), post-transcriptionally control gene expression (Chuang and Jones, 2007). MiRNAs’ expression is dynamic, acting in several cellular pathways, and one single miRNA can target multiple genes whereas several miRNA can target the same gene (Gambari et al., 2011; Zhang et al., 2012). Indeed, miRNAs, can function as oncogenes or tumor suppressors (Rupaimoole and Slack, 2017), impacting on metabolic pathways, including glutaminolysis, glycolysis and Krebbs cycle (Chuang and Jones, 2007; Chen et al., 2012).”

A correction has been made to ***Epigenetic Mechanisms in Cancer: a Brief Overview*, subsection *Histone Modifying Enzymes***, paragraph four:

“Gene expression regulation by epigenetics mechanisms is very adaptative to environmental factors (Feil and Fraga, 2012). As cancer cells divide, acquired epigenetics states may be maintained through cell division by DNA methylation, repressive chromatin, or gene regulatory circuits, giving rise to adaptive epi-clones that fuel malignant progression (Flavahan et al., 2017). By contrast, permissive or plastic states may allow oncogene activation or non-physiologic cell fate transitions. This plasticity state may confer advantage for cancer cells and be selected as drivers. In mutated gliomas, particularly those with IDH mutation, chromatin structure destabilizes and, thereby, triggers epigenetic instability. Thus, hypermethylated phenotype associated to IDH mutant gliomas promote aberrant activation of platelet-derived growth factor receptor A (PDGFRA), which fosters uncontrolled proliferative signalling, a recognized hallmark of cancer (Flavahan et al., 2017).”

A correction has been made to ***Tumor Metabolism***, paragraph seven:

“Metabolic rewiring in cancer profoundly affects gene expression regulation. Although Metabolite profiles profoundly impact epigenetic regulation, although genetic impact is minimal (Reid et al., 2017). Therefore, epigenetic and metabolic alterations in cancer cells are closely mechanistically linked. The accessibility to epigenetic enzymes’ co-factors might be altered due to reprogramming of cell metabolism, which gives rise to metabolic by-products that affect enzymatic activity, altering the epigenetic profile of cancer cells (Sharma and Rando, 2017). Moreover, metabolism is affected by altered expression of key enzymes due to epigenetic changes, impacting on control of several metabolic pathways (Wong et al., 2017). Thus, several malignant traits are supported by the interplay between metabolomics and epigenetics, promoting neoplastic transformation. An integrative comprehension of epigenetic and metabolic interplay in cancer is far from complete, but conceptual schemes are starting to emerge.”

A correction has been made to ***Epigenetic Regulation of Metabolic Enzymes in Cancer***, paragraph two:

“Conversely, glucose transporter 1 (GLUT1) overexpression is due to derlin-3 promotor hypermethylation, implicated in GLUT1 proteasome degradation (Lopez-Serra et al., 2014). Moreover, metabolic reprogramming of cancer cells derives from oncogene activation. The PI3K/AkT/mTOR pathway together with MYC and HIF1α transcription factors have been implicated in glycolytic metabolism (Jang et al., 2013). Concomitantly, PTEN (Salvesen et al., 2001; Kang et al., 2002; Soria et al., 2002; Garcia et al., 2004; Alvarez-Nunez et al., 2006), VHL (Herman et al., 1994; Schmitt et al., 2009; Vanharanta et al., 2013) and PDH (Place et al., 2011), which repress this pathway, are epigenetically silenced through promoter methylation, resulting in PI3K/AkT/mTOR pathway constitutive activation.”

A correction has been made to ***Epigenetic Regulation of Metabolic Enzymes in Cancer***, paragraph four:

“Among HDACs, Sirtuins’ family (HDAC class III) has been the most extensively studied concerning cell metabolism regulation. Indeed, SIRT6 and SIRT3 have been implicated in glucose homeostasis regulation (Chalkiadaki and Guarente, 2015). Glycolytic metabolism and glutaminolysis depending on HIF1α and MYC, respectively, are abrogated by SIRT6 (Zhong et al., 2010; Sebastian et al., 2012). Accordingly, SIRT6 deletion, observed in different tumours, like colon, pancreatic and hepatocellular carcinomas, leads to increased H3K9ac levels resulting in glycolytic gene expression upregulation promoting cellular transformation and, consequently tumour growth and progression (Sebastian et al., 2012; Chalkiadaki and Guarente, 2015). Additionally, mitochondrial SIRT3 was also shown to regulate the glucose homeostasis in HIF1α-dependent manner (Bell et al., 2011). In fact, SIRT3 loss is associated with cellular metabolism shift towards enhanced glycolysis in cancer cells [168]. Furthermore, SIRT4 suppresses tumour growth by repressing glutamine metabolism (Jeong et al., 2013). Specifically, SIRT4 overexpression inhibits glutamine utilization and proliferation by a MYC-dependent manner in human Burkitt lymphoma cells (Jeong and Haigis, 2015). Nevertheless, SIRT4 downregulation has been reported in several tumours, like bladder, gastric and breast cancer (Chalkiadaki and Guarente, 2015). Although less consistently than SIRT4, SIRT1 was also associated with tumour suppressor function in cellular metabolic regulation (Chalkiadaki and Guarente, 2015), repressing glycolytic metabolism, indirectly through HIF1α deacetylation and directly by inhibiting the glycolytic enzyme phosphoglycerate mutase 1 (PGAM1) through deacetylation (Lim et al., 2010). Interestingly SIRT1 also has been implicated in lipid metabolism regulation under tumour nutrient deprivation (Jeong and Haigis, 2015). Regarding SIRT2, both oncogene or tumour suppressor functions have been suggested, depending on the tumour context (Chen et al., 2013; McGlynn et al., 2014). SIRT2 deacetylases FOXO1, modulating glucose and lipid metabolism in cellular stress and caloric restriction conditions (Jeong and Haigis, 2015). Indeed, SIRT2 promotes gluconeogenesis by deacetylating the enzyme phosphoenolpyruvate carboxykinase (PEPCK) (Jiang et al., 2011). SIRT2 is also involved in tumour metabolism regulation through MYC stabilization by deacetylating H4K16ac (Liu et al., 2013). Thus, SIRT2 and MYC are implicated in tumour metabolism regulation of MYC-induced malignancies working as a positive feedback loop.”

A correction has been made to ***Metabolites and Cancer Epigenetic Landscape Interplay*, subsection* Metabolites and DNA/Histone Methylation***, paragraph one:

“DNA methylation is the most extensively studied epigenetic alteration in cancer, which typically affects promoter regions of cancer-related genes, leading to transcriptional repression (Kulis and Esteller, 2010). Unlike acetylation, histone methylation does not affect chromatin ionic charge, but functions as docking site for recruiting specific proteins/transcription factors. Histone methylation is an epigenetic mark associated with transcriptional repression or activation depending on the type of residue and the number of methyl group (Greer and Shi, 2012). In both cases, activity is dependent of S-adenosyl-methionine (SAM), a methyl donor product of serine-glycine one-carbon metabolism and methionine cycle, which is synthetized from ATP and methionine by the enzyme methionine adenosyltransferase (MAT) (**Figure 4**). SAM provides methyl groups which consistently release S-adenosyl-homocysteine (SAH), a potent inhibitor of DNMTs and HMTs (Mentch et al., 2015; Wong et al., 2017). Thus, SAM/SAH cellular ratio is a major determinant of chromatin methylation (**Figure 4**). In fact, increased SAM/SAH ratio associates with tumour suppressor genes’ hypermethylation and inappropriate silencing, whereas decreased SAM/SAH ratio contributes to reduced methylation at oncogenes’ promoters (Wong et al., 2017).”

A correction has been made to ***Metabolites and Cancer Epigenetic Landscape Interplay*, subsection *Metabolites and DNA/Histone Methylation***, paragraph two:

“Glycine-N-methyltransferase (GNMT) is involved in SAM levels’ homeostasis. Indeed, GNMT deficiency was associated with RASSF1 and SOCS2 promoter methylation, and oncogenic pathways activation in hepatocellular carcinoma (Mudd et al., 2001; Martinez-Chantar et al., 2008). Additionally, aberrant expression of Nicotinamide N-methyltransferases (NMMT, a limiting enzyme that metabolizes SAM) has been observed in lung, liver, kidney, bladder and colon cancers (Wu et al., 2008; Tang et al., 2011; Thomas et al., 2013; Zhang et al., 2014). Cell lines overexpressing NMMT display significantly decreased HMTs activity and, consequently, histone methylation marks, especially at H3K4, H3K9, H3K27 and H4K20, associating with more aggressive/pluripotent phenotype. Conversely, because DNMTs have lower Km values for SAM compared to HMTs, DNA methylation is not affected by aberrant NMMT expression levels (Wong et al., 2017). Moreover, amino acid transporters overexpression by cancer cells may directly increase methionine uptake (Fuchs and Bode, 2005; Haase et al., 2007). Likewise, serine is also in high demand by cancer cells, contributing to increased ATP availability in cancers cells and provision of SAM, which is synthesized from methionine (Rabhi et al., 2017). Interestingly, increased methylthioadenosine (MTA) concentration in cancer cells harbouring 5-methylthioadenosine phosphorylase (MTAP) deletions results in decreased H4R3me2 mark and, consequently, arginine methyltransferase 5 (PRMT5) inhibition (Kryukov et al., 2016).”

A correction has been made to ***Metabolites and Cancer Epigenetic Landscape Interplay*, subsection* Metabolites and DNA/Histone Methylation***, paragraph three:

“DNA and histone methylation is also regulated by DNA and histone demethylases. TET proteins catalyse 5-methyl-cytosine (5-mC) oxidation, generating 5-hydroxymethyl-cytosine (5-hmC), allowing for demethylation of aberrantly methylated cytosine residues (Huang and Rao, 2014). Histone demethylases dependent of flavin (LSD1) and Jumonji C-domain-containing (JMJD) enzymes demethylate lysine marks (Dimitrova et al., 2015). Metabolites may serve as substrate and/or co-factors for DNA and histone demethylases. TCA cycle generates several intermediary metabolites, some of which involved in DNA/histone demethylases activity. Concerning histone demethylation reaction catalysed by LSD1, it is accomplished by reduction of co-factor FAD^+^ to FADH_2_ at mitochondrial level. LSD1 demethylase activity appears to control metabolism favouring *de novo* fatty acids synthesis over gluconeogenesis in hepatocytes and adipocytes (Zheng et al., 2015). In tumour cells, LSD1 overexpression leads to methyl group removal from H3K9me and H3K4me, favouring tumour progression, cell proliferation and stemness (Hino et al., 2016).”

A correction has been made to ***Metabolites and Cancer Epigenetic Landscape Interplay*, subsection* Metabolites and DNA/Histone Methylation***, paragraph four:

“Both JMJDs and TETs are dioxygenases dependent of α-ketoglutarate (α-KG) as co-factor (**Figure 4**), being inhibited by TCA cycle intermediates succinate and fumarate (Xiao et al., 2012): α-KG is produced from isocitrate by mitochondrial enzymes isocitrate dehydrogenase 2 (IDH2) and 3 (IDH3) as an intermediary of TCA cycle. In addition to isocitrate, α-KG is also synthetized from amino acids such as arginine, glutamine, histidine and proline (**Figure 4**) (Etchegaray and Mostoslavsky, 2016; Rabhi et al., 2017; Kim and Yeom, 2018). The α-KG/succinate ratio regulates chromatin status in embryonic stem cells (ESCs) through JMJD3 and Tet1/Tet2 demethylation of H3K9me3, H3K27me3 and H4K20me (Carey et al., 2015). Although Jmj-KDMs expression deregulation has been reported in various cancers, how fluctuations in α-KG correlate with Jmj-KDM-driven cancers is not completely understood. In melanoma cells, glutamine depletion at hypoxic tumour core associates with histone hypermethylation, mostly H3K27 methylation, due to reduced α-KG. H3K27 methylation is particularly increased in genes associated with cancer cells dedifferentiation and confers resistance to BRAF^V600E^ inhibitors (Pan et al., 2016). Finally, α-KG is an important metabolite for activity of other dioxygenases, including RNA N^6^-methyladenosine (m^6^A) demethylation and EglN prolyl-4-hydroxylation (Schvartzman et al., 2018).”

A correction has been made to ***Metabolites and Cancer Epigenetic Landscape Interplay*, subsection* Metabolites and Histone Acetylation***, paragraph two:

“Acetyl-CoA is a central metabolite coordinating the activity of HATs, since increased levels contribute to increased histone acetylation (**Figure 4**) (Lee and Workman, 2007). This metabolite is a key intermediary produced during catabolism and anabolism, both in mitochondria and cytoplasm, associating with breakdown of carbohydrates and fats, *via* glycolysis and β-oxidation, respectively (Pietrocola et al., 2015). Additionally, acetyl-CoA might derive from ketone bodies and amino acids (Pietrocola et al., 2015). In mitochondria, pyruvate generated from glycolysis and β-oxidation is converted to acetyl-CoA. As a mitochondrial impermeable metabolite, citrate produced in Krebs cycle from acetyl-CoA is transported to cytoplasm and subsequently converted to acetyl-CoA through ATP-citrate lyase (ACL) (**Figure 4**) (Wellen et al., 2009; Zaidi et al., 2012; Kim and Yeom, 2018). Additionally, when glucose availability is limited and/or in hypoxia conditions, acetate may be a source of acetyl-CoA. Acetate that enters the mitochondria is used to acetyl-CoA synthesis through mitochondrial acetyl-CoA synthetase 2 (AceCS2) or promotes acetyl-CoA production in cytoplasm through acetyl-CoA synthetase 2 (AceCS1) (**Figure 4**) (Kim and Yeom, 2018). Acetyl-CoA levels are quite dynamic and directly dependent of nutrient availability. Indeed, histone acetylation is regulated by acetyl-CoA absolute levels and the ratio acetyl-CoA/coenzyme A in cancer cells (Lee et al., 2014).The expression of ACL and the availability of citrate modulate cellular acetyl-CoA levels. In colorectal cancer, ACL silencing suppressed histone acetylation (Wellen et al., 2009) whereas ACL overexpression was reported in different tumours (Migita et al., 2008), probably contributing for nuclear acetyl-CoA pool, necessary for histone acetylation and glycolytic enzymes expression.”

A correction has been made to ***Metabolites and Cancer Epigenetic Landscape Interplay*, subsection* Metabolites and Histone Acetylation***, paragraph three:

“The glycolytic flux and mitochondrial citrate production, subsequently migrating to cytosol and nucleus is promoted by metabolic reprogramming in cancer cells. In pancreatic adenocarcinoma, Akt signalling activation, through activated KRAS^G12D^, promotes nuclear acetyl-CoA accumulation and ACL phosphorylation, inducing histone acetylation (Lee et al., 2014). Additionally, MYC increases mitochondrial export of acetyl-groups and upregulates HAT-GCN5 expression, inducing H4 acetylation (Knoepfler et al., 2006). After glucose, glutamine is the main acetyl-CoA source in tumour cells. In glucose deprivation, glutamine is used as substrate and acetyl-CoA production in TCA cycle favours histone acetylation, stimulating tumour cell proliferation and growth (Le et al., 2012; Lu and Thompson, 2012; McDonnell et al., 2016; Rabhi et al., 2017).”

A correction has been made to ***Metabolites and Cancer Epigenetic Landscape Interplay*, subsection* Metabolites and Histone Acetylation***, paragraph five:

“The antagonistic functions of HDACs and HATs regulate histone acetylation. Lysine/histone deacetylases (KDAC/HDAC) catalyse removal of the acetyl group from lysine residues of histones, favouring condensed chromatin status and consequent gene transcriptional repression (Yoshida et al., 2017). HDACs are divided in four classes according to structural and mechanistic similarities: zinc-dependent class II, II and IV (classical HDACs) and NAD^+^ dependent class III (sirtuins’ family) (Seto and Yoshida, 2014).”

A correction has been made to ***Metabolites and Cancer Epigenetic Landscape Interplay*, subsection* Metabolites and Histone Acetylation***, paragraph six:

“Deacetylation reactions are also metabolic responsive. In addition to the well-known HDAC inhibitors (class I, II and IV), trichostatin (TSA) and suberoylanilide hydroxamic acid (SAHA), HDAC activity can be antagonized by different cellular metabolites (**Figure 4**) (Marchion and Munster, 2007). Butyrate, a short fatty acid, used as energy source for colon cell growth, inhibits class I, II and IV HDAC activity (Candido et al., 1978; Fan et al., 2015). Additionally, in breast cancer cell lines, ketogenic bodies, namely β- hydroxybutyrate, were shown to reduce the activity of class I and II HDACs (Martinez-Outschoorn et al., 2012). Furthermore, lactate has been shown to inhibit HDAC activity in cancer cells, similar to TSA and butyrate (Latham et al., 2012; Wagner et al., 2015). In cancer cells, histone deacetylation mediated by HDACs causes tumour suppressor genes silencing (Nakagawa et al., 2007). Metabolic reprogramming can affect histone acetylation by accumulation of metabolites that inhibit histone deacetylases. In colon cancer cells, Warburg effect leads to accumulation of butyrate due to suppression to acetyl-CoA conversion. Consequently, increased butyrate inhibits HDAC activity, upregulating pro-apoptotic genes. When glycolytic metabolism is inhibited, butyrate promotes acetyl-CoA production facilitating colon cancer cell growth. Thus, metabolic reprogramming can instruct cancer cells to distinctively utilize metabolites to mediate differential epigenetic modifications (Donohoe et al., 2012). Remarkably, a similar effect was also observed with β-hydroxybutyrate in tumour brain cells. This metabolite is produced from ketogenesis and used as energy source by normal brain cells. Upregulated glycolytic rates suppress conversion in tumour cells, resulting in β-hydroxybutyrate accumulation which inhibits histone deacetylation (Newman and Verdin, 2014). Furthermore, in tumour cells, enhanced glycolytic phenotype increases lactate production that is exported to tumour microenvironment. This cellular lactate may negatively regulate HDAC activity and, consequently, gene expression. Interestingly, in breast cancer cells, lactate induced a distinctive gene expression signature related with stemness (Martinez-Outschoorn et al., 2011). Thus, HDAC inhibition by lactate might be involved in cancer cell fate decision.”

A correction has been made to ***Metabolites and Cancer Epigenetic Landscape Interplay*, subsection* Metabolites and Histone Acetylation***, paragraph seven:

**“**NAD^+^ is an important cofactor for histone deacetylases class III (sirtuins) activity. This important redox co-factor is required by many enzymes involved in catabolic or oxidative pathways including glycolysis, TCA cycle and β-oxidation of fatty acids (**Figure 4**) (Imai and Guarente, 2014). NAD^+^ levels determines sirtuins activity, depending on nutrient availability. When energy is in excess, NAD^+^ is depleted, generating lower NAD^+^/NADH ratio, inhibiting sirtuins’ activity (Li and Kazgan, 2011; Canto et al., 2015). In contrast, NAD^+^ levels rise in energy deficiency situations, like physical exercise or caloric restriction (increased NAD^+^/NADH ratio leading to AMPK activation), entailing sirtuins’ activation (Canto et al., 2009). SIRT1 and SIRT6 are overexpressed in those conditions contributing to decreased histone (H3K9ac and H3K14ac) acetylation (Etchegaray and Mostoslavsky, 2016). In parallel, decreased glycolytic gene expression and increased gluconeogenesis gene expression also occurs, promoting cell survival (German and Haigis, 2015). Cancer cells rely on glycolysis even in the presence of oxygen, leading to low NAD^+^/NADH ratio and consequent inhibitory effect on sirtuins’ activity. Moreover, deviant gene transcription due to increased histone acetylation is caused by augmented activity of HATs (acetyl-CoA induced) and sirtuins, favouring tumour growth and progression (Wong et al., 2017). Indeed, SIRT6 was reported as tumour suppressor in pancreatic cancer (Kugel et al., 2016), as well as other isoforms in different tumours. Furthermore, in colorectal cancer, SIRT6 expression loss associated with glycolytic genes upregulation, promoting cellular transformation, tumour growth and aggressiveness (Sebastian et al., 2012). Thus, sirtuins may suppress tumorigenesis through epigenetic mechanisms that modulate metabolic reprogramming.”

A correction has been made to ***Metabolites and Cancer Epigenetic Landscape Interplay*, subsection* Oncometabolites and Epigenetic Regulation***, paragraph one:

“Some metabolites are able to promote tumorigenesis by altering the epigenome, being defined as oncometabolites (Nowicki and Gottlieb, 2015). These oncometabolites, namely fumarate, succinate and 2-hydroglutarate, are generated in excess due to mutations in TCA cycle-associated enzymes. Mutations in genes encoding metabolic enzymes result in pathological accumulation of metabolites that may affect histone and DNA methylation. IDH1 and IDH2 mutations have been identified in acute myelogenous leukemia, lymphoma, glioblastoma, chondrosarcoma and other solid tumours (Yan et al., 2009; Ward et al., 2010; Cairns et al., 2012; Cancer Genome Atlas, 2012). These loss-of-function mutations in IDH1/2 prevent conversion of α-KG to isocitrate, favouring synthesis of 2-hydroxyglutarate (2-HG), instead (**Figure 4**) (Dang et al., 2009). This oncometabolite is competitive inhibitor of α-KG, inhibiting TET and JmjC activity (**Figure 4**) (Xu et al., 2011). Moreover, 2-HG is also increased in breast (Terunuma et al., 2014) and renal cancer (Shim et al., 2014). 2-HG is the product of malate dehydrogenase 1 and 2, and LDHA, which has been linked with deficiency of L-2-hydroxyglutarate dehydrogenase and activation of MYC, in renal and breast cancer, respectively. Interestingly, the enantiomer S-2-HG is produced by LDHA under hypoxic conditions, also affecting histone methylation and hypoxic transcriptional responses (Intlekofer et al., 2015). *In vitro* enzymatic assays showed that 2-HG inhibits Tet1/2 activity, abrogating 5hmC formation in human cell lines (Figueroa et al., 2010). Additionally, IDH R132H mutant cells display CpG island methylator phenotype, similarly to gliomas and acute myeloid leukemia, with reduced Tet1/2 activity (Figueroa et al., 2010; Turcan et al., 2012).”

A correction has been made to ***Epigenetic-Metabolism Crosstalk in Cancer Cells as a Therapeutic Target*, subsection* Tumor Metabolism Inhibitors***, paragraph one:

“In cancer cells, increased histone acetylation is, in part, caused by the elevated glycolytic flow (and associated flux of glucose), mediated by acetyl-CoA and citrate. Thus, glycolysis inhibition may lead to histone acetylation modulation. 2-Deoxyglucose (2-DG), a glucose analog may competitively inhibit G6P production, hindering the glycolytic pathway (Chen and Gueron, 1992). Furthermore, 2-DG treatment suppresses acetyl-CoA levels, leading to global histone H3 and H4 decrease in several cancer cell lines and associates with compromised DNA repair and cancer cells sensitization to DNA-damaging agents (Liu et al., 2015). Another glycolysis inhibitor, 3-bromopyruvate, decreases acetyl-CoA and induces differentiation in embryonic stem cells (Moussaieff et al., 2015).”

A correction has been made to ***Epigenetic-Metabolism Crosstalk in Cancer Cells as a Therapeutic Target*, subsection* Tumor Metabolism Inhibitors***, paragraph two:

“Several inhibitors targeting glutaminase (GLS) (which deaminates glutamine to glutamate) have been developed. Compounds 968 and CB-839 are two GLS inhibitors. In breast cancer cells, decreased expression of several cancer-associated genes was observed as a result of alterations in H3K4 methylation and H4K16 acetylation due to 968 (Simpson et al., 2012), whereas CB-839 is currently in Phase I trial in solid and hematological cancers (Robinson et al., 2007; Wang et al., 2010).”

A correction has been made to ***Epigenetic-Metabolism Crosstalk in Cancer Cells as a Therapeutic Target*, subsection* Tumor Metabolism Inhibitors***, paragraph three:

“IDH mutations are key events in epigenetic landscape of leukemias and gliomas. IDH1/2 inhibition has been suggested to suppress 2-HG production. In mutant IDH glioma cells, AGI-5198 was shown to inhibit 2-HG production and cell growth, inducing H3K9me3 and H3K27me3 demethylation, not affecting DNA methylation. (Rohle et al., 2013). The same was reported in human IDH mutant chondrosarcoma cells (Li et al., 2015). Subsequently, novel mutant IDH1^R132H^ inhibitors, including AG-120, AG-881, ML309, GSK321 and GSK864 have shown efficacy (Wong et al., 2017). Additionally, AG-221, a first-in-class inhibitor of mutant IDH2, leads to 2HG reduction in IDH2 mutant leukemia and survival benefit in primary human IDH2 mutant AML xenografts (Yen et al., 2017). This IDH2 inhibitor underwent Phase I and Phase II clinical trials, in which effective 2HG levels decrease was observed both in bone marrow and in plasma, achieving sustainable remission of disease in some patients with advanced hematologic malignancies harbouring IDH2 mutations (Wong et al., 2017). Likewise, AGI-6780, another mutant IDH2 inhibitor, caused demethylation of DNA and histones, reversing gene expression patterns that were acquired during tumorigenesis owing to epigenetic deregulation (Wang et al., 2013).”

A correction has been made to ***Epigenetic-Metabolism Crosstalk in Cancer Cells as a Therapeutic Target*, subsection* Tumor Metabolism Inhibitors***, paragraph four:

“Because SAM availability is critical for DNMTs and HMTs activity and SAH hydrolase is essential for methylation homeostasis maintenance, SAH hydrolase inhibitors have emerged, namely DZNep (3-deazaneplanocin A) (Glazer et al., 1986). DZNep was ineffective in reactivating silenced genes due to promoter methylation in cancer cells, although it globally inhibited DNA and histone methylation, reactivating a subset of developmental genes. However, a synergistic effect against leukemic cells was observed when combined with the DNMT inhibitor 5-aza-2’-deoxycytidine (5-Aza), through activation of genes silenced by histone and DNA methylation (Momparler et al., 2014; Momparler and Cote, 2015).”

A correction has been made to ***Epigenetic-Metabolism Crosstalk in Cancer Cells as a Therapeutic Target*, subsection *Epigenetic Enzymes’ Inhibitors***, paragraph one:

“Inhibition of DNMTs effectively reverses DNA methylation and two inhibitors (5-Aza and 5-azacytidine) were approved by the American and European regulatory agencies for treatment of selected hematological maligancies. In solid tumors, results from clinical trials were less effective and the effect of these inhibitors on cancer metabolism is currently unknown. Nevertheless, in IDH mutant cancers inhibitors of DNMTs were able to reverse DNA methylation. Treatment of IDH1 mutant glioma cells suppressed tumour growth and was effective in inducing differentiation compared to mutant IDH inhibitors (Borodovsky et al., 2013; Turcan et al., 2013).”

A correction has been made to ***Epigenetic-Metabolism Crosstalk in Cancer Cells as a Therapeutic Target*, subsection* Epigenetic Enzymes***’ Inhibitors, paragraph two:

“Furthermore, evidence that inhibition of HDAC affects the metabolism of cancer cells is growing. Colorectal cancer cell line HT29 treated with a combination of butyrate and TSA (both HDAC inhibitors) disclosed reduced glycolytic metabolism (Alcarraz-Vizan et al., 2010). In a different tumour model (multiple myeloma), the HDAC inhibitors vorinostat and valproate treatment effectively abrogated the expression of GLUT1 and HKI activity (Wardell et al., 2009). Exposure of H460 lung cancer cell line to butyrate and TSA resulted in a reversal of the glycolytic phenotype, with transition to dependency from oxidative phosphorylation (Amoedo et al., 2011) and a similar effect was disclosed in breast cancer cells (Rodrigues et al., 2015). Thus, effective inhibition of HDAC activity may reverse aerobic glycolysis in cancer. Because sirtuin family members, play an important role in metabolic regulation of cancer cells, especially SIRT6, specific inhibitors may provide an additional strategy to target cancer cell metabolism (Feldman et al., 2013).”

A correction has been made to the ***Conclusions***, paragraph one:

“Altered metabolism and epigenetic deregulation have mutual influence in adaptation of cancer cells to a constantly changing environment. Metabolic rewiring in cancer cells affects the epigenome facilitating tumour development and progression. Specifically, acetyl-CoA pools are key in epigenetic control. Depending on metabolic pathway involved in acetyl-CoA production, histone acetylation patterns in different transcriptional gene targets may engage. Thus, the specificity of the metabolite-driven epigenetic regulation of targets is important to allow better understanding of cancer biology. Additionally, identification of transcription factors activated in different metabolic states, as well as the role of metabolic enzymes in nuclear compartment, will allow for discovery of mechanisms underlying integration of metabolic signalling in chromatin.”

A correction has been made to the ***Conclusions***, paragraph two:

“Most available data on epigenetic and metabolic crosstalk in cancer cells derives from 2D cell culture models, which do not realistically portray the complexity of this interaction *in vivo*, especially when the critical role of TME is considered. In fact, epigenetic drugs may have limited success in solid tumours with extensive hypoxic regions (Braiteh et al., 2008; Chu et al., 2013), which has been associated not only with tumour progression and aggressiveness but also with therapy resistance (Wilson and Hay, 2011). Moreover, hypoxic tumour cells display epigenetic abnormalities, namely DNA hypomethylation and histone hyperacetylation (Johnson et al., 2008). Thus, approaches that target epigenetic mechanisms should consider the impact of both tumour microenvironment and metabolism.”

A correction has been made to the ***Conclusions***, paragraph three:

“Previous studies have demonstrated that inhibition of epigenetic factors (e.g., HDACs, DNMTs) has an impact in cancer cell metabolism, although further studies are required to fully understand its effectiveness and the underlying mechanisms. Furthermore, clinical trials should incorporate biomarker analysis to unravel epigenomic and metabolomic markers allowing for identification of patient subsets that may benefit most from metabolic/epigenetic modulators treatment. Additionally, combining epigenetic and metabolic targeting might provide a more effective means of inhibiting tumour progression. Overall, in view of the tumour microenvironment’s key role in epigenetic plasticity, patients might also benefit from inclusion of other therapeutic strategies that target TME components (e.g., anti-angiogenics, immune checkpoint inhibitors), as well as conventional chemotherapy. Altogether and from a theoretical standpoint, these combinations are likely to positively impact on cancer patients’ management.”

The authors apologize for this overlap and state that these corrections do not change the scientific conclusions of the article in any way. The original manuscript has been updated.

